# Differential induction of mutant SOD1 misfolding and aggregation by tau and α-synuclein pathology

**DOI:** 10.1186/s13024-018-0253-9

**Published:** 2018-05-18

**Authors:** Michael C. Pace, Guilian Xu, Susan Fromholt, John Howard, Benoit I. Giasson, Jada Lewis, David R. Borchelt

**Affiliations:** 1 Department of Neuroscience, Center for Translational Research in Neurodegenerative Disease, McKnight Brain Institute, University of Florida, 1275 Center Drive, BMS Building J-491, PO Box, Gainesville, FL 32610-0244 USA; 2 SantaFe Healthcare Alzheimer’s Disease Center, Gainesville, FL USA

**Keywords:** Proteostasis, Protein misfolding, Tau, α-Synuclein, SOD1, Proteinopathy

## Abstract

**Background:**

Prior studies in *C. elegans* demonstrated that the expression of aggregation-prone polyglutamine proteins in muscle wall cells compromised the folding of co-expressed temperature-sensitive proteins, prompting interest in whether the accumulation of a misfolded protein in pathologic features of human neurodegenerative disease burdens cellular proteostatic machinery in a manner that impairs the folding of other cellular proteins.

**Methods:**

Mice expressing high levels of mutant forms of tau and α-synuclein (αSyn), which develop inclusion pathologies of the mutant protein in brain and spinal cord, were crossed to mice expressing low levels of mutant superoxide dismutase 1 fused to yellow fluorescent protein (G85R-SOD1:YFP) for aging and neuropathological evaluation.

**Results:**

Mice expressing low levels of G85R-SOD1:YFP, alone, lived normal lifespans and were free of evidence of inclusion pathology, setting the stage to use this protein as a reporter of proteostatic function. We observed robust induction of G85R-SOD1:YFP inclusion pathology in the neuropil of spinal cord and brainstem of bigenic mice that co-express high levels of mutant tau in the spinal axis and develop robust spinal tau pathology (JNPL3 mice). In contrast, in crosses of the G85R-SOD1:YFP mice with mice that model spinal α-synucleinopathy (the M83 model of αSyn pathology), we observed no G85R-SOD1:YFP inclusion formation. Similarly, in crosses of the G85R-SOD1:YFP mice to mice that model cortical tau pathology (rTg4510 mice), we did not observe induction of G85R-SOD1:YFP inclusions.

**Conclusion:**

Despite robust burdens of neurodegenerative pathology in M83 and rTg4510 mice, the introduction of the G85R-SOD1:YFP protein was induced to aggregate only in the context of spinal tau pathology present in the JNPL3 model. These findings suggest unexpected specificity, mediated by both the primary protein pathology and cellular context, in the induced “secondary aggregation” of a mutant form of SOD1 that could be viewed as a reporter of proteostatic function.

**Electronic supplementary material:**

The online version of this article (10.1186/s13024-018-0253-9) contains supplementary material, which is available to authorized users.

## Background

Neurodegenerative diseases such as Alzheimer’s disease (AD), Parkinson’s disease (PD), frontotemporal dementia (FTD), amyotrophic lateral sclerosis (ALS) and Huntington’s disease are often defined pathologically by the accumulation of misfolded proteins that become aggregated to form intracellular and/or extracellular inclusions [1–4]. This underlying theme across these diseases has suggested that a similar pathogenic mechanism contributes, at least in part, to the development and/or progression of these disorders. To date, defects in many cellular pathways have been implicated in the pathogenesis of these diseases [5, 6]. Some of these suspected pathways are critical in cellular protein quality control such as the ubiquitin-proteasome system (UPS) (reviewed in [7]) and autophagy (reviewed in [8]). Indeed, it has been previously demonstrated that pathological forms of tau (associated with Alzheimer’s disease and various tauopathies) and αSyn (associated with Parkinson’s disease and other synucleinopathies) can hinder the efficacy of the proteasome [9–16]. This finding is further supported by the accumulation of ubiquitin-positive inclusions in the cases of many neurodegenerative disorders [17–19]. Hence, one hypothesis that has gained traction is that protein aggregation in these neurodegenerative disorders is a biomarker of underlying dysfunction in protein quality control systems (reviewed in [20]).

Protein homeostasis (proteostasis) is maintained by a network of factors (reviewed in [21–23]) that primarily fall into three main components at the cytosolic level: molecular chaperones responsible for folding newly synthesized proteins and refolding misfolded proteins [24], the UPS which is responsible for the degradation of misfolded and inherently short-lived proteins (reviewed in [25, 26]), and the autophagy-lysosomal pathway which is necessary for the removal of large insoluble protein aggregates that cannot otherwise be degraded [27]. The implications of this systemic malfunction in proteostasis could be widespread at the cellular level, and one particular idea that has emerged is the concept of “secondary” protein misfolding, where the accumulation of one misfolded protein imposes a burden on the proteostasis network that leaves other vulnerable proteins with insufficient support to fold correctly [28, 29]. Disruption of the proteostasis network could potentially explain the origin of mixed pathologies in human neurodegenerative diseases, which are relatively common [30–34]. Accordingly, we have previously shown the aggregation of phosphorylated TDP-43 protein as a secondary event to the aggregation of phosphorylated tau in two independent transgenic models [35]. These instances of mixed pathologies could, however, have been a result of cross-seeding, which has been demonstrated for many proteins pathologically associated with neurodegeneration [36–41].

The original concept of secondary misfolding was characterized in the invertebrate *C. elegans*. In this study, it was found that the expression of an aggregation-prone protein could impair the folding integrity of other proteins, or “bystander” proteins [28]. Specifically, a fragment of (exon 1) mutant human huntingtin (*HTT*) gene containing a polyglutamine (polyQ) expansion was co-expressed with temperature-sensitive (TS) mutant forms of paramyosin and dynamin-1. Both proteins achieve functional conformations at lower temperatures (15 °C), but are inactive at 25 °C. Mutant huntingtin exon-1 fragments are very prone to aggregate [42] and, when expressed in the muscle wall of *C. elegans* concomitantly with these TS mutants, the TS proteins failed to achieve active conformations at 15 °C [28]. This outcome was thought to be due to the stress placed upon the proteostasis network by mutant huntingtin, overwhelming the system and preventing proteins that are particularly dependent upon the proteostasis network (e.g. TS mutant proteins) from achieving active conformations [28]. Interestingly, the added burden of co-expressed TS mutant proteins exacerbated the aggregation of mutant huntingtin, supporting the argument that the capacity of the cellular protein folding machinery of *C. elegans* is limited and easily over-burdened.

In the present study, we asked whether the deposition of human tau or αSyn aggregates in the central nervous system (CNS) of mouse models might impose a burden on proteostatic function using a mutant form of SOD1 fused to YFP as a reporter in a paradigm akin to the foregoing *C. elegans* studies. We have been using a mouse model that expresses the G85R variant of SOD1 fused to YFP as model in studies of prion-like propagation of misfolded SOD1 [43–45]. Hemizygous mice expressing G85R-SOD1:YFP do not intrinsically develop ALS symptoms or show inclusion pathology, while homozygous G85R-SOD1:YFP mice develop paralysis from 6 months onward with spinal cords that contain fluorescent inclusions and detergent-insoluble G85R-SOD1:YFP [46]. Thus, the hemizygous G85R-SOD1:YFP mouse could be viewed as model that is sub-threshold for induction of disease. In such a setting, any perturbation that diminished proteostatic function could then lead to a breach of threshold to induce mutant SOD1 aggregation. Importantly, the YFP tag on the G85R-SOD1 protein allows for simple detection and visualization of aggregation and inclusion formation, and we use this feature as a readout to assess secondary misfolding in mice that develop tau and αSyn pathology. We crossed the G85R-SOD1:YFP mice to three different models of proteinopathies: 1) a model of spinal tau pathology expressing human P301L tau (termed JNPL3 [47]), 2) a model of spinal αSyn pathology expressing human A53T αSyn (termed M83 [48]), and 3) a model of cortical tau pathology expressing human P301L tau (termed rTg4510 [49, 50]). Despite abundant proteinopathy in these models, only bigenic mice from the cross with JNPL3 mice caused robust G85R-SOD1:YFP pathology to develop. Our findings demonstrate complex interactions between pathologically misfolded tau, αSyn and the proteostatic network in triggering the “secondary aggregation” of our mutant SOD1 reporter.

## Methods

### Transgenic mice

To model tauopathy, we utilized both the JNPL3 and rTg4510 mouse models. Briefly, JNPL3 mice (maintained on the Swiss Webster background from Taconic) express mutant human tau (P301L, 4R0N) under the mouse prion promoter which leads to mutant tau pathology primarily in the spinal cord and brainstem (though other regions are more modestly affected) [47]. rTg4510 mice (maintained on a hybrid 129S6/FVB background) are bigenic mice that express both human tau with the P301L mutation (4R0N) behind by a disrupted minimal CMV promoter and the tet-transactivator (tTA) driven by a Ca^2+^ calmodulin kinase II (CaMKII) promoter (forebrain-specific). The tTA protein binds to the disrupted promoter to drive mutant tau expression at high levels, primarily within the hippocampus and neocortex [49, 50]. To model α-synucleinopathy, we used the M83 mouse model (maintained on the hybrid C3H/B6 background). This model overexpresses mutant (A53T) human αSyn under the mouse prion promoter [48], and develops αSyn pathology primarily within the spinal cord, brainstem midbrain, hypothalamus, thalamus and periaqueductal gray regions (with other brain regions also somewhat affected), resulting in a severe motor phenotype and paralysis. This pathology and phenotype occurs between 8 and 16 months of age in homozygous M83 mice, but later than 21 months in hemizygous M83 mice [48]. Lastly, hemizygous mice (maintained on the FVB background) expressing the G85R mutant of SOD1 tagged to YFP under the human SOD1 promoter were used for all crossing experiments [46].

All mice were kept in specific pathogen free cages prior to harvesting and histology procedures. All animals were handled and processed according to approved protocols by the University of Florida Institutional Animal Care and Use Committee (IACUC). All applicable international, national, and/or institutional guidelines for the care and use of animals were followed.

### Breeding scheme to generate mice co-expressing G85R-SOD1:YFP and mutant proteins associated with human proteinopathies

Mice heterozygous for the G85R-SOD1:YFP transgene were bred to mice transgenic for either mutant tau or αSyn. In order to generate JNPL3-G85R-SOD1:YFP animals, we first bred heterozygous JNPL3 mice to heterozygous G85R-SOD1:YFP mice to produce male animals expressing both the JNPL3 tau and SOD1 transgenes. These were bred to a homozygous JNPL3 female mice to produce offspring that were then bred to generate a large cohort of mice, some of which expressed both mutant tau and our reporter G85R-SOD1:YFP transgene. A subset of these mice were expected to be homozygous for the tau transgene and to develop early onset tauopathy, with a subset of these mice being transgenic for the G85R-SOD1:YFP transgene. To generate mice expressing both mutant αSyn and our G85R-SOD1:YFP reporter, homozygous M83 mice were bred to mice heterozygous for the G85R-SOD1:YFP transgene. Mice expressing G85R-SOD1:YFP as well as the transgenes associated with the rTg4510 line are triple transgenic animals, which were generated by first crossing mice transgenic for P301L mutant tau to mice transgenic for G85R-SOD1:YFP. The double transgenic mice generated from this cross were then crossed to mice transgenic for tTA under the CaMKII promoter in order to generate rTg4510 mice (tau/tTA) expressing G85R-SOD1:YFP.

### Intramuscular human αSyn fibril injections into hemizygous M83 and bigenic M83-G85R-SOD1:YFP transgenic mice to seed αSyn pathology

As has been previously described [51], sonicated wild type human αSyn fibrils (2 mg/mL) were injected at a volume of 5 μL into hemizygous M83xG85R-SOD1:YFP mice once they reached 8 weeks of age. Injections were conducted using a Hamilton 10 μL syringe (Reno, NV) along with a 25-gauge needle. The needle was injected ~ 1 mm deep into the gastrocnemius muscle bilaterally in each animal. Mice were anesthetized with isoflurane during the procedure.

### Tissue processing, immunohistochemistry and image microscopy

Mice were euthanized by isoflurane anesthesia overdose with exsanguination and transcardial perfusion with cold PBS. The harvested brains were bisected sagittally and for a subset of animals, one hemi-brain was frozen on dry ice (stored at − 80 °C) and the other was drop fixed in 4% paraformaldehyde for 48 h. For a subset of the animals, both hemi-brains were drop-fixed with one used for cryostat sections (10 μm) to directly visualize fluorescence and the other was embedded in paraffin for sectioning (5 μm). The harvested spinal cords were divided into 4 equivalent segments. For two of the segments, the spinal column was drop-fixed in 4% paraformaldehyde for 48 h before embedding in paraffin for sectioning. In a subset of animals, spinal segments were flash frozen on dry ice and then stored at − 80 °C. For a subset of animals, all 4 segments of spinal column were drop-fixed in 4% paraformaldehyde so that 2 of the segments could be sectioned by cryostat with 2 segments embedded in paraffin for sectioning. All sections were attached to Superfrost Plus microscope slides (Fisher Scientific, Hampton, NH) for imaging. To prepare paraffin-embedded tissue for histology, sections first were de-paraffinized and rehydrated through immersion in serial dilutions of ethanol. For sections used for direct fluorescence microscopy, the frozen section or rehydrated paraffin section was coverslipped with Vectashield (Vector Laboratories, Burlingame, CA) mounting medium. Tissue sections used for immunohistochemistry were rinsed in water, followed by antigen retrieval via a 30-min incubation in a steamer containing citrate buffer (10 mM sodium citrate with 0.05% Tween-20, pH 6.0). The citrate antigen retrieval procedure quenches the fluorescence of the G85R-SOD1:YFP, requiring the use of a primary antibody to GFP/YFP for visualization. Tissue sections were blocked using a PBS solution containing 3% normal goat serum and 0.1% Triton X-100. Primary antibodies in 3% normal goat serum in PBS-T were incubated overnight at 4 °C. For DAB-mediated immunostaining, endogenous peroxidases were blocked using a solution of 0.3% H_2_O_2_ in PBS. An ABC kit (Vector Laboratories, Burlingame, CA) was used with a DAB reagent set (KPL, Gaithersburg, MD) to detect signal. Sections were then counterstained with hematoxylin prior to dehydration in ethanol and coverslipping. Primary antibodies used included AT8 (1:500, mouse monoclonal, Thermo Fisher, Waltham, MA), MC1 (1:125, mouse monoclonal, Peter Davies), PHF1 (1:500, mouse monoclonal, Peter Davies), GFP (green fluorescent protein)/YFP (1:200, rabbit polyclonal, Invitrogen, Waltham, MA) and JL-8 GFP/YFP (1:200, mouse monoclonal, Clontech, Mountain View, CA, USA). Secondary fluorescent antibodies used for immunofluorescence staining included goat anti-rabbit IgG (1:1000, Alexa Fluor 488, Invitrogen, Waltham, MA) and goat anti-mouse IgG (1:1000, Alexa Fluor 568, Invitrogen, Waltham, MA). Biotinylated secondary antibodies (Vector Laboratories, Burlingame, CA) were used for DAB-mediated staining for 30 min at room temperature (1:500). Tissue sections were imaged using an Olympus DSU-IX81 spinning disc confocal microscope (Tokyo, Japan) or scanned using the Scanscope FL image scanner (Aperio, Vista, CA).

### Fluorescence quantification and statistical analysis

Fluorescence quantification, when necessary, was conducted using ImageJ (version 1.51 g). Statistical analyses were conducted using GraphPad PRISM (version 7.0 h, La Jolla, CA), as indicated in applicable figures.

### Preparation of brain and spinal cord tissues for immunoblot analysis

To assess the levels of G85R-SOD1:YFP in the brains of trigenic rTg4510 x G85R-SOD1:YFP mice, one hemi-forebrain was homogenized in PBS (10% weight/volume) with 1% protease inhibitor cocktail P8340 in DMSO (Sigma Aldrich, St. Louis, MO). Protein concentrations were measured by the BCA (bicinchoninic) assay (Fisher Scientific, Hampton, NH) and 20 μg of protein from each brain homogenate was adjusted to 1 x Laemmli buffer, boiled, loaded onto a 4–20% Tris-glycine gel (Invitrogen, Waltham, MA) and subjected to SDS-PAGE (120 V for 90 min). Proteins were then transferred to nitrocellulose membranes overnight at 100 mA.

To generate detergent insoluble fractions from JNPL3/G85R-SOD1:YFP mice, G85R-SOD1:YFP mice, JNPL3 mice, and nontransgenic control mice, we used a method used to fractionate insoluble SOD1 aggregates that has been previously described [52, 53]. Briefly, spinal cord tissues were dissected from the frozen spinal columns after a brief thaw and homogenized in 1× TEN (10 mM Tris-HCl pH 7.5/1 mM EDTA/100 mM NaCl) at a 10:1 volume to weight ratio. This homogenate was then mixed 1:1 with a second buffer containing 1× TEN with 1% NP40 and 1% protease inhibitor cocktail P8340 (Sigma Aldrich, St. Louis, MO). This mixture was sonicated and centrifuged at > 1,000,000 *g* in an airfuge (Beckman Coulter, Brea, CA) for 10 min to separate soluble from insoluble protein fractions. The pellet fraction was then washed in a buffer containing 1× TEN with 0.5% NP40, sonicated, and spun down to obtain the final pellet fraction which was resuspended in 30 μL of buffer containing 1× TEN with 0.5% NP40, 0.25% SDS, 0.5% deoxycholate and 1% protease inhibitor cocktail. Protein concentrations in each fraction were measured by BCA (bicinchoninic) assay (Fisher Scientific, Hampton, NH). 20 μg of protein from each fraction, suspended in 1× Laemmli buffer, was boiled and then electrophoresed into 16% Tris-glycine gels before transfer to nitrocellulose membranes overnight at 100 mA. The membranes were then analyzed with antibodies to SOD1 as described below.

To examine the levels of insoluble αSyn and SOD1 in M83/G85R-SOD1:YFP mice, the protocol used for detergent extraction and sedimentation differed slightly, following a previously described method [54]. Briefly, the tissue was initially homogenized in PBS containing 1% protease inhibitor cocktail and then centrifuged at 100,000 x g for 30 min. The supernatant was collected and saved as the PBS-soluble fraction. The pellet was resuspended in 1× TEN buffer containing 0.5% NP40 by brief sonication and then centrifuged at 100,000 x g (Optima L100 K Ultracentrifuge [Beckman Coulter, Brea, CA] using a 70.1 Ti rotor at 35,000 RPM) for 30 min to produce NP-40 soluble and insoluble fractions. The NP-40 insoluble pellet was then resuspended in a volume of TEN with 2% sodium deoxycholate equal to the original homogenization volume by brief sonication. 30 μL of either PBS-soluble or NP40-insoluble protein fractions were mixed with 4 x Laemmli buffer, boiled, and loaded onto a 16% Tris-glycine gel. After transfer to nitrocellulose at 300 mA for 1 h, the membranes were probed with antibodies to SOD1 and αSyn as described below.

### Immunoblotting and western blot quantification

Nitrocellulose membranes were blocked in a 5% powdered milk (Nestle Carnation, Glendale, CA) solution in PBS-T. The membrane was then incubated in primary antibody in the blocking solution overnight, followed by three 10-min washes in PBS-T. Primary antibodies included GAPDH (1:5000, Meridian Life Science, Memphis, TN), Tau13 (1:1000, BioLegend, San Diego, CA), mouse/human SOD1 (1:4000, generated in-house) and human SOD1 (1:2500, generated in-house [55]). Horseradish peroxidase-conjugated secondary anti-mouse or anti-rabbit antibody (Vector Laboratories, Burlingame, CA) was used in order to visualize proteins by chemiluminescence using the Pierce ECL Western Blot Substrate Kit (Thermo Scientific, Waltham, MA). Western blot quantification was conducted using GeneTools by Syngene (in correlation with the GeneSys imager, Daly City, CA).

## Results

### Mutant tau induces G85R-SOD1:YFP inclusion pathology in the spinal cord and brainstem

In order to determine whether tau pathology could induce G85R-SOD1:YFP to aggregate, we analyzed the JNPL3 transgenic mice model of spinal tau pathology on the hemizygous G85R-SOD1:YFP background. For this study, only female bigenic JNPL3/G85R-SOD1:YFP mice were analyzed due to the differences in tau transgene expression between males and females that we previously reported in the JNPL3 mice (specifically that females express mutant tau at much higher levels than their male counterparts) [56]. JNPL3/G85R-SOD1:YFP and JNPL3 transgenic animals were harvested at humane endpoints (ranging from 7 to 15.5 months of age), characterized by paralysis in at least one limb (usually a hind limb). Compared to littermates that were transgenic only for tau, JNPL3/G85R-SOD1:YFP mice showed a statistically significant, but modest acceleration of the paralysis phenotype (Additional file 1: Figure S1).

**Fig. 1:**
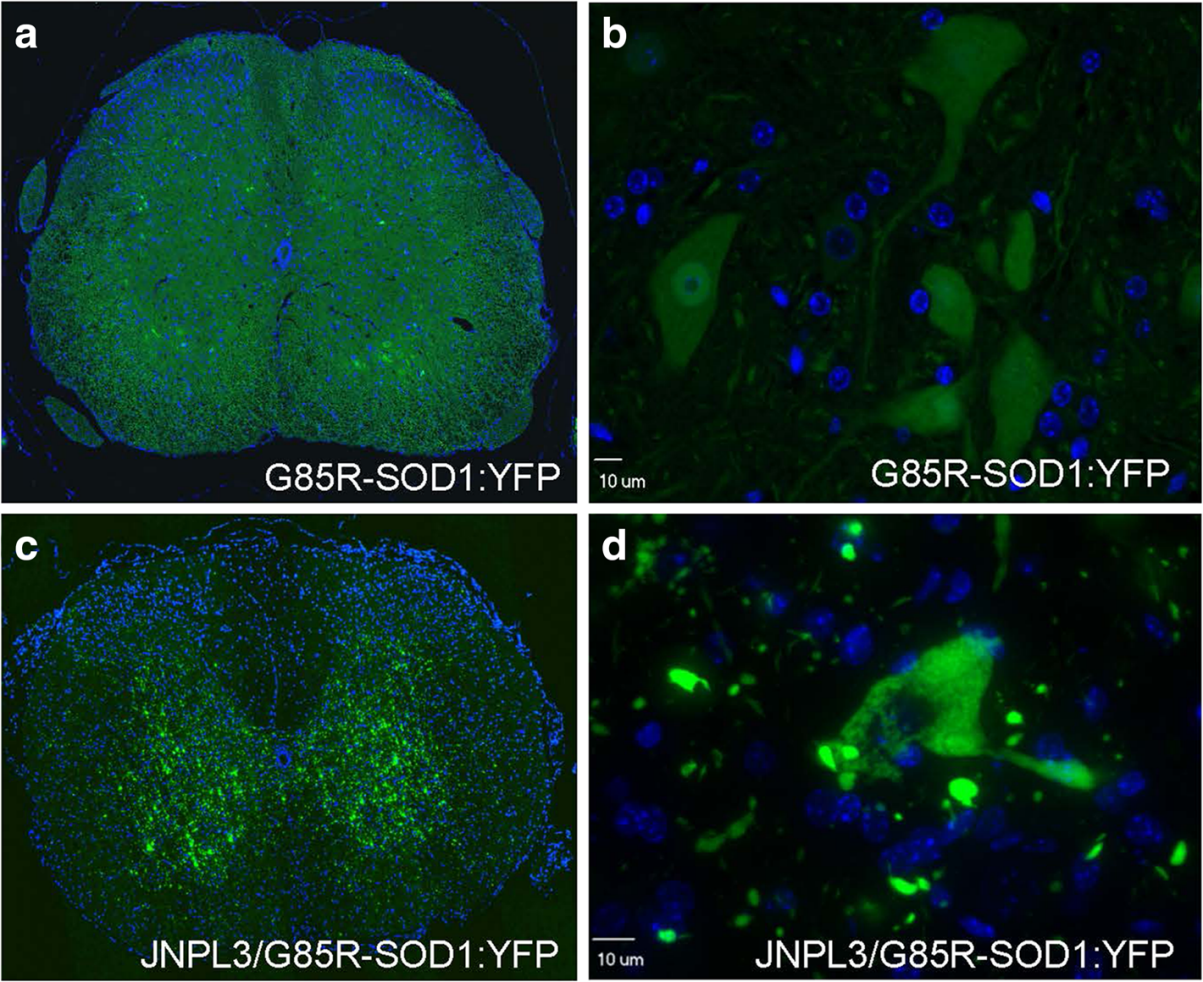
G85R-SOD1:YFP aggregation into punctate inclusions within the spinal cord of JNPL3/G85R-SOD1:YFP mice. Compared to the diffuse distribution of G85R-SOD1:YFP in spinal motor neurons of single transgenic animals (**a** and **b**), the fluorescence is organized into large neuropil inclusions with granular/punctate accumulation in the cell bodies of spinal motor neurons of bigenic JNPL3/G85R-SOD1:YFP mice (**c** and **d**). Exposure times were kept consistent across images and set to capture images of the inclusions in the bigenic mice at optimal exposure. Nuclei were stained with DAPI (blue). All animals analyzed were female. Representative images (40× magnification) of the ventral horn within the spinal cord are shown for 8 JNPL3-G85R-SOD1:YFP double transgenic mice and 3 G85R-SOD1:YFP single transgenic mice (aged 7 to 15.5 months). An additional low power image of the spinal ventral horn bigenic JNPL3/G85-SOD1:YFP mice is provided in Additional file 2: Figure S2a

We hypothesized that the presence of tau pathology in the JNPL3/G85R-SOD1:YFP animals could act as a stressor upon cellular proteostasis that could cause bystander aggregation of G85R-SOD1:YFP and the formation of fluorescent inclusions. In contrast to the soluble and diffuse distribution of G85R-SOD1:YFP seen in hemizygous animals (Fig. 1a and b), JNPL3/G85R-SOD1:YFP bigenic mice exhibited robust inclusion pathology in the form of widespread punctate aggregates in the gray matter visible by direct fluorescence (Fig. 1c and d). This pathology was predominantly observed in the spinal cord and brainstem (Fig. 1, Additional file 2: Figure S2), the same regions that are subject to heavy tau burden in the JNPL3 model (Additional file 3: Figure S3, spinal tauopathy shown) [47]. The abundant fluorescent neuropil aggregates with granular/punctate pathology in the cell body was similar in appearance to what has been described for the homozygous G85-SOD1:YFP mice that develop paralysis [46] and for G85R-SOD1:YFP mice induced to develop paralysis by prion-like transmission experiments [44].

To further confirm that G85R-SOD1:YFP in the bigenic tau/SOD1 mice was aggregated, we conducted detergent extraction and sedimentation of spinal cords from these mice to determine changes in protein solubility. In previous study, we have demonstrated that the aggregates formed by mutant SOD1 become aberrantly crosslinked through disulfide oxidation and to visual these aggregates the SDS-PAGE was performed in the absence of reducing agent [53]. As expected from this prior study, soluble G85R-SOD1:YFP was detected in spinal cord extracts from bigenic tau/SOD1 mice and mice expressing G85R-SOD1:YFP alone, but NP40-insoluble, highly crosslinked, G85R-SOD1:YFP was only detected in the tau/SOD1 bigenic mice that showed inclusion pathology (Additional file 4: Figure S4a and b). Collectively, these data demonstrate that the G85R-SOD1:YFP protein was induced to misfold and aggregate in the spinal cords of mice with abundant spinal tau pathology.

### Localization of SOD1 pathology relative to tau pathology

**Fig. 2:**
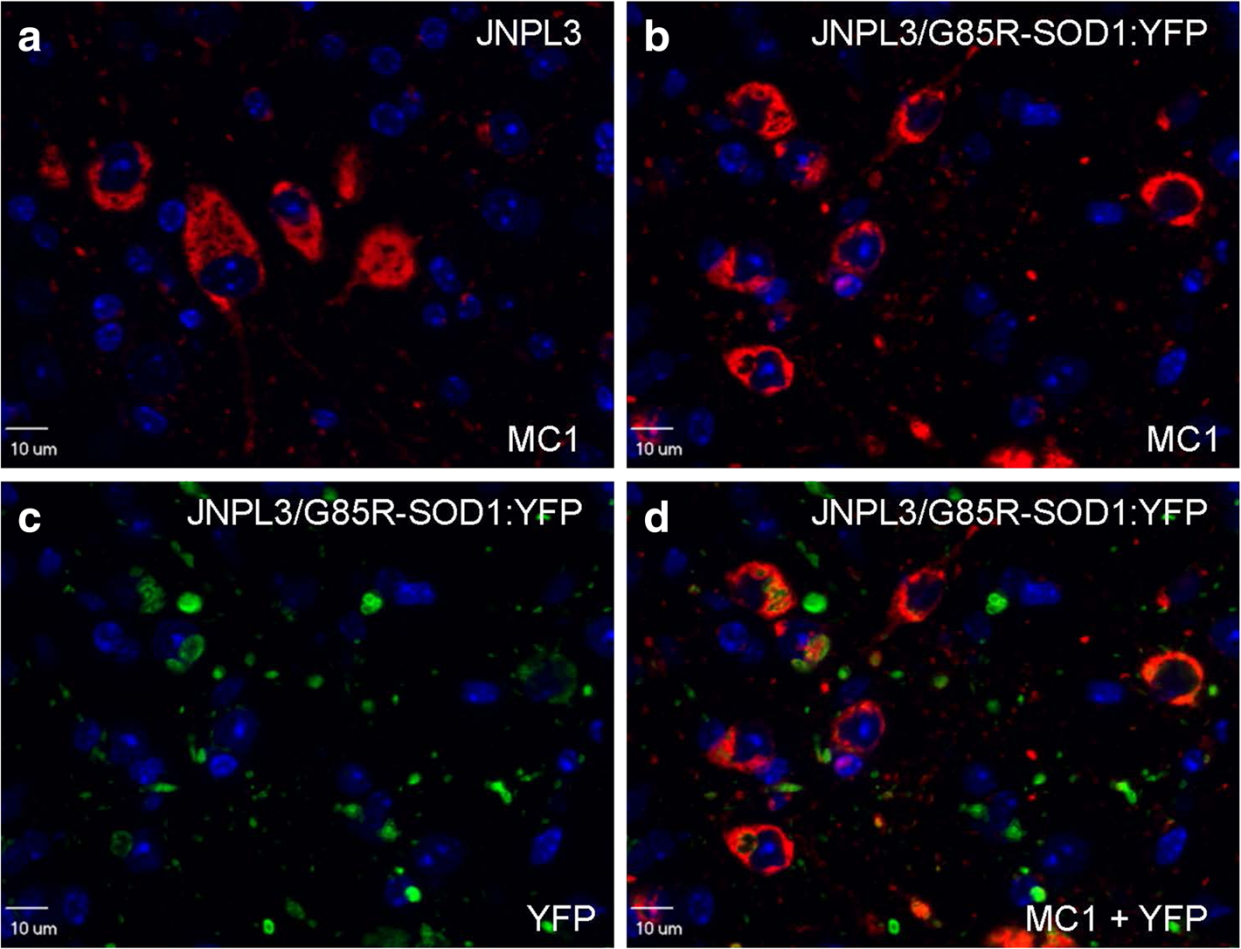
Localization of tau MC1 immunoreactivity versus G85R-SOD1:YFP pathology in bigenic JNPL3/G85R-SOD1:YFP mice. Misfolded human tau recognized by the MC1 antibody (red) appears similar in JNPL3 tau-transgenic versus JNPL3/G85R-SOD1:YFP bigenic animals (**a**, **b**). G85R-SOD1:YFP pathology, detected by an YFP antibody (green) (**c**), does not robustly co-localize with tau pathology in double transgenic animals (**d**). Nuclei were stained with DAPI (blue). Representative images (60× magnification) are shown within the ventral horn of the spinal cord of 8 JNPL3 and 8 JNPL3/G85R-SOD1:YFP animals. All animals analyzed were female aged 7 to 15.5 months

**Fig. 3:**
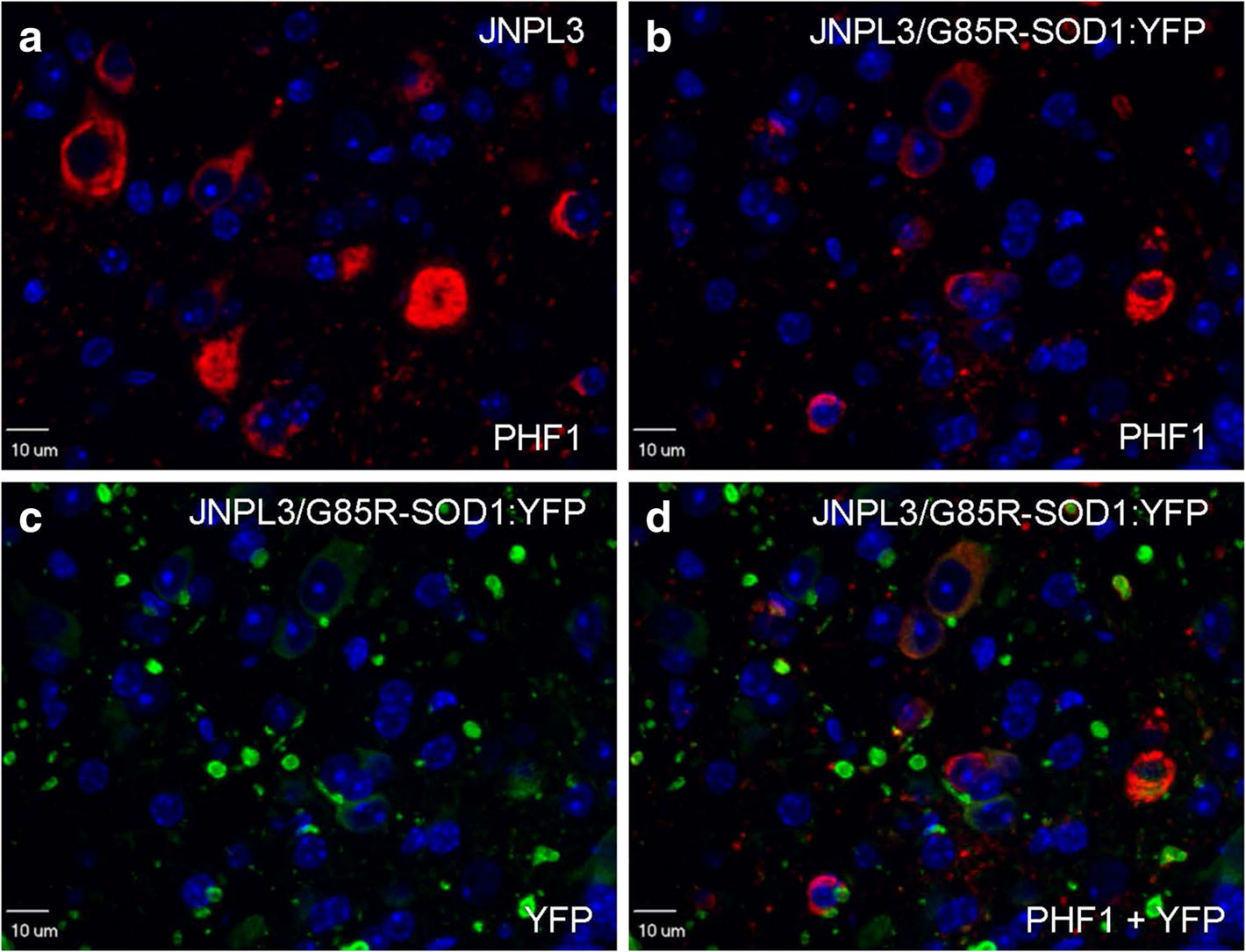
Localization of phosphotau immunoreactivity versus G85R-SOD1:YFP pathology in bigenic JNPL3/G85R-SOD1:YFP animals. Hyperphosphorylated human tau pathology recognized by the PHF1 (Ser396/Ser404; red) antibody appears similar in JNPL3 tau-transgenic versus JNPL3/G85R-SOD1:YFP bigenic mice (**a**, **b**). G85R-SOD1:YFP pathology, detected using an YFP antibody (green) (**c**), does not robustly co-localize with tau pathology in double transgenic animals (**d**). Nuclei were stained with DAPI (blue). Images are of 60× magnification within the ventral horn of the spinal cord of JNPL3 and JNPL3/G85R-SOD1:YFP mice. The images shown are representative of 8 JNPL3 and 8 JNPL3/G85R-SOD1:YFP female animals aged 7 to 15.5 months

We next sought to determine whether the G85R-SOD1:YFP inclusions were simply co-depositing with aggregating tau. To our knowledge, no interaction between these two proteins has been reported, nor has SOD1 pathology been described as a secondary event to tauopathy. We performed double immunostaining of brain and spinal cord tissues with three well-characterized tau antibodies (MC1, PHF1, or AT8) in conjunction with a primary antibody to YFP to assess tau and SOD1 pathology (Figs. 2 and 3, Additional file 5: Figure S5). Both end-stage JNPL3 mice and JNPL3/G85R-SOD1:YFP mice had extensive tau pathology (Figs. 2a-b, 3a-b; Additional files 3 and 5: Figures S3, S5a-b). Consistent with our original reports on the pathology of JNPL3 mice, both hyperphosphorylated tau as detected by AT8 and PHF1 immunostaining, and tau of an abnormal conformation as detected by MC1 immunostaining were present [47, 56]. We observed limited co-localization between the tau and SOD1 aggregates in the spinal cord of JNPL3/G85R-SOD1:YFP bigenic animals (Figs. 2d and 3d; Additional file 5: Figure S5d). Overall, bigenic animals exhibit both robust tau and SOD1 pathology, but with no obvious direct evidence for co-aggregation occurring between the two proteins.

### Lack of G85R-SOD1:YFP inclusion pathology induction in the rTg4510 model of cortical and hippocampal tauopathy

**Fig. 4:**
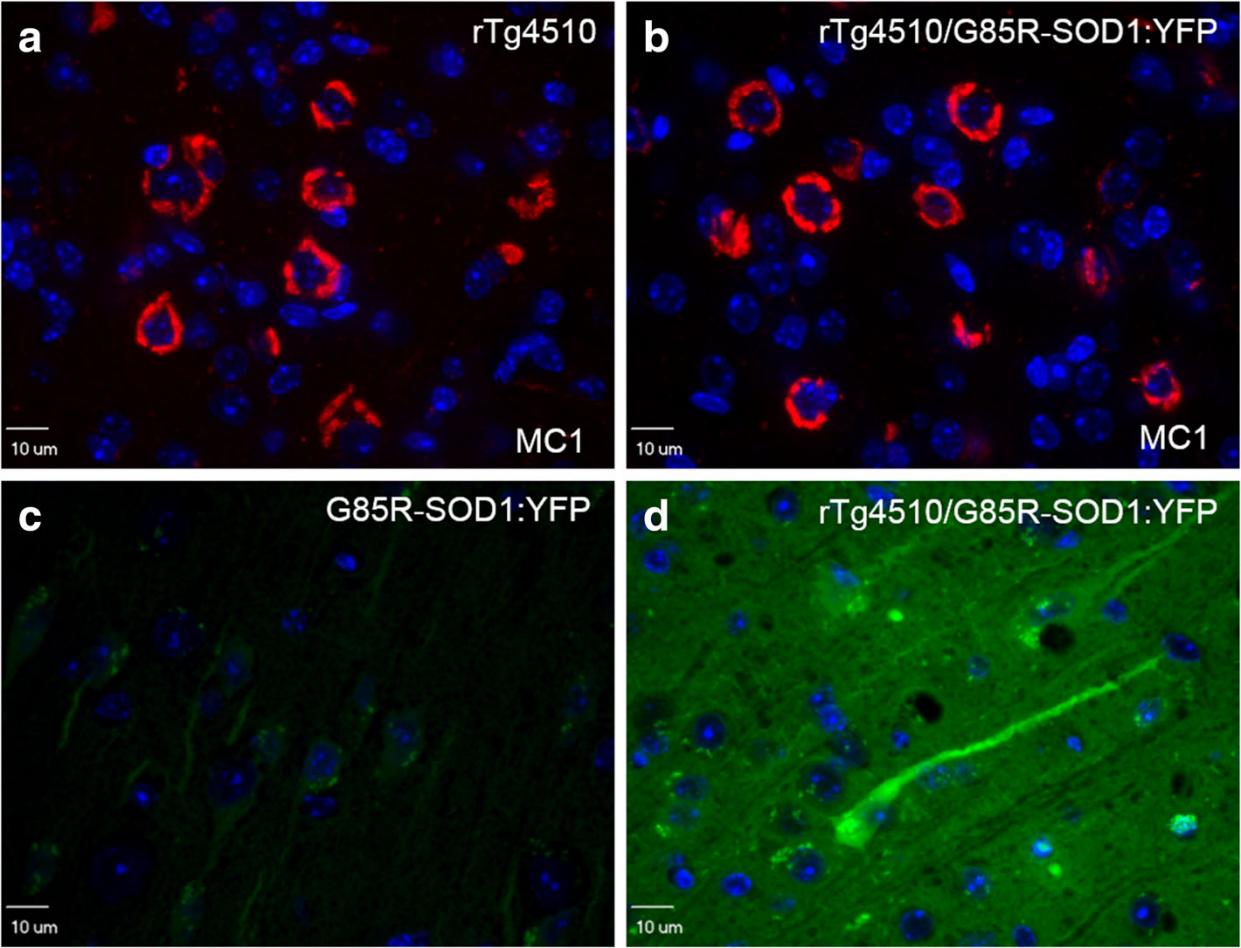
G85R-SOD1:YFP does not form inclusions in the forebrain of rTg4510/G85R-SOD1:YFP bigenic. The severity of neurofibrillary tangle pathology in the hippocampus and cortex (**a**, cortex shown immunostained with the MC1 antibody, red) in rTg4510 mice is similar to that of rTg4510/G85R-SOD1:YFP bigenic mice (**b**). Although the intensity of YFP fluorescence in the trigenic P301L/G85R-SOD1:YFP mice was higher than that of mice expressing G85R-SOD1:YFP alone, but there was no evidence of organization into inclusion structures (**c** and **d**; see Additional file 6: Figure S6). There were isolated cells that were hyperfluorescent (**d**), but the fluorescence in these cells did not appear to be organized into fibrils. All images were taken at 60X magnification of 5 μm paraffin-embedded sections. Nuclei were stained with DAPI (blue). Exposure time and specifications were kept consistent across all images, optimized to the rTg4510/G85R-SOD1:YFP tissue sections. Representative images are shown for 5 rTg4510/G85R-SOD1:YFP mice (1 male, 4 female) and 4 G85R-SOD1:YFP mice (4 males) between 8 and 9 months of age

Neither homozygous nor hemizygous G85R-SOD1:YFP mice typically develop inclusion pathology in the forebrain (cortex, hippocampus, striatum) similar to the degree that is seen in the spinal cord [46]. To assess whether cortical/hippocampal tau pathology could also induce secondary aggregation of G85R-SOD1:YFP, we used the rTg4510 model that develops robust cortical tau pathology by 5.5 months of age (Fig. 4a-b; Additional file 6: Figure S6) [49, 50]. The introduction of G85R-SOD1:YFP expression in rTg4510 mice yielded no noticeable motor phenotype and despite very robust tau pathology, there was no clear formation of widespread fluorescent inclusions in rTg4510/G85R-SOD1:YFP mice (Fig. 4d, cortex shown). In these trigenic mice, there was a general increase in overall fluorescence intensity throughout the cortex and hippocampus with occasional cells that were intensely fluorescent (Fig. 4d; for quantification of direct fluorescence see Additional file 7: Figure S7) as compared to single transgenic G85R-SOD1:YFP controls (Fig. 4c-d). However, we could not attribute the increased fluorescence to increased levels of G85R-SOD1:YFP protein in immunoblot analysis of forebrains in the trigenic mice compared to G85R-SOD1:YFP alone (Additional file 7: Figure S7) and thus the basis for the heightened fluorescence intensity is unknown. In any case, there was no evidence of fluorescent inclusions as was observed in the JNPL3/G85R-SOD1:YFP bigenic mice.

### Paucity of induced G85R-SOD1:YFP aggregation in the M83 model of αSynucleinopathy

**Fig. 5:**
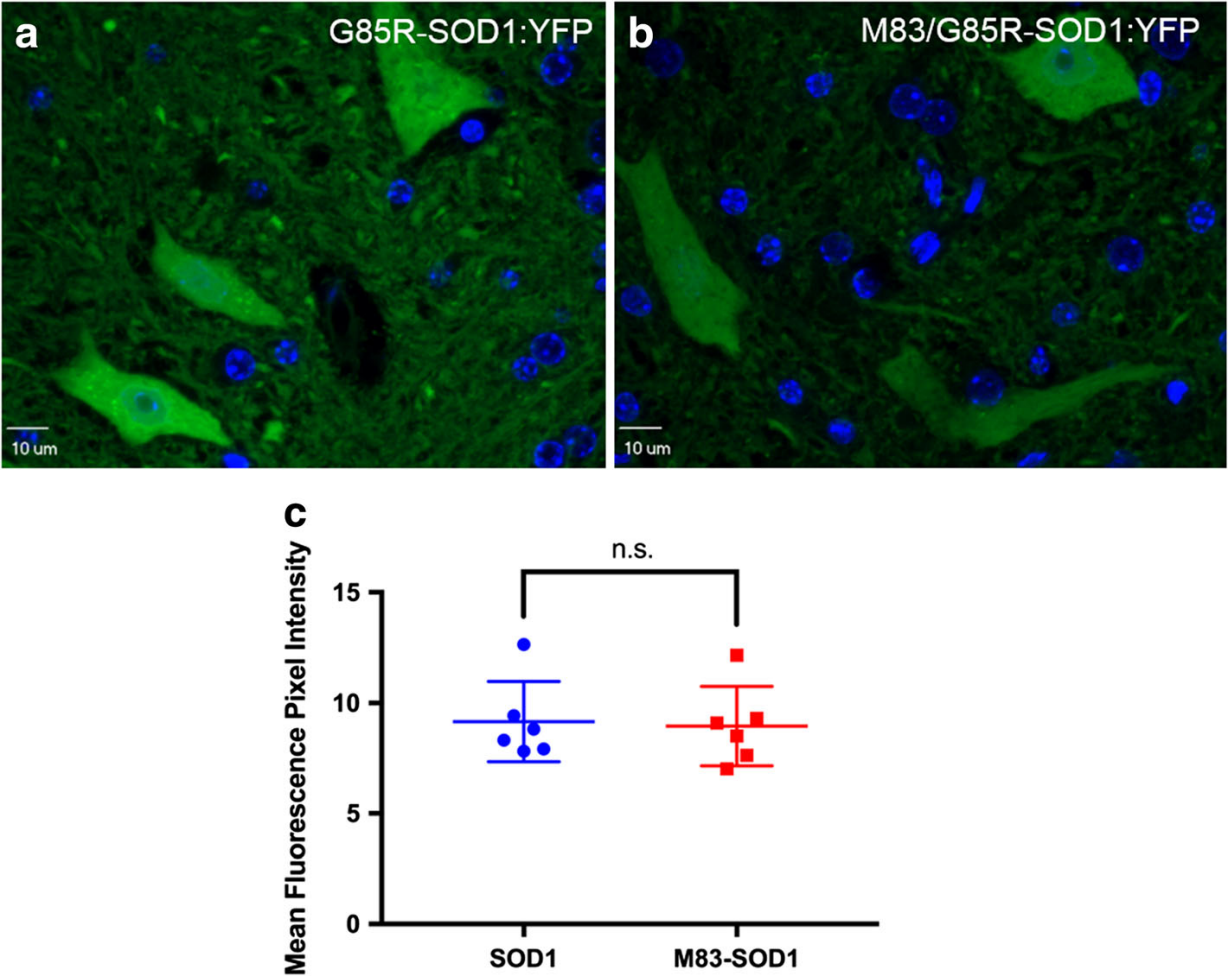
Lack of G85R-SOD1:YFP inclusion pathology in the spinal cord of M83/G85R-SOD1:YFP mice. Mice were IM injected to induce αSyn pathology at 2 months of age and then euthanized at a humane endpoint (both hind limbs paralyzed). Compared to the diffuse distribution of G85R-SOD1:YFP in single transgenic animals (**a**), M83 mice expressing the G85R-SOD1:YFP reporter protein have a similar distribution of the protein (**b**), visible by direct fluorescence. Exposure times were kept consistent across images. Nuclei were stained with DAPI (blue). Representative images (60× magnification) of the ventral horn within the spinal cord are shown for 8 M83/G85R-SOD1:YFP double transgenic mice (5 female, 3 male) and 6 G85R-SOD1:YFP single transgenic mice (2 female, 4 male). Mean fluorescence intensity was not statistically significant between M83 mice expressing G85R-SOD1:YFP (abbreviated M83-SOD1) and those expressing G85R-SOD1:YFP alone (abbreviated SOD1) (**c**). Statistical analysis was conducted using GraphPad Prism (version 7.0 h). Error bars show mean ± S.D.; unpaired T-test

Given our observation of aggregated SOD1:YFP reporter in the JNPL3 model of spinal tauopathy, we next sought to determine whether the effects we observed were specific to tau or could be extended to a different type of spinal proteinopathy. We utilized the M83 model of αSyn-opathy that expresses mutant (A53T) αSyn under the mouse prion promoter [48], which is the same vector that was used to create the JNPL3 tauopathy model. The two models show very similar anatomical pathological burden (compare Additional file 3: Figure S3 to Additional file 8: Figure S8) and both develop paralytic phenotypes. To induce the αSyn pathology, bigenic M83/G85R-SOD1:YFP mice were injected intramuscularly with fibrillized human αSyn protein as previously described [51]. This seeded induction of αSyn pathology produces a predictably accelerated robust spinal pathology that is accompanied by a paralytic motor phenotype (between 3 and 4 months post-IM-injection) compared to uninjected hemizygous M83 mice, which acquire this phenotype later than 21 months of age [48, 51]. Unexpectedly, the spinal cords and brainstems of paralyzed bigenic M83/G85R-SOD1:YFP lacked any evidence of G85R-SOD1:YFP inclusions (Fig. 5). There was no significant difference observed in motor phenotype for mice expressing only αSyn versus double transgenic animals expressing both αSyn and G85R-SOD1:YFP (Additional file 9: Figure S9). The level of αSyn pathology in these bigenic mice was not obviously different from that of M83 littermates that also were IM injected with αSyn fibrils (see Additional file 8: Figure S8). Thus, in stark contrast to tau pathology, we do not observe secondary G85R-SOD1:YFP pathology in the presence of αSyn pathology.

To confirm the pathological findings, we assessed the levels of soluble and insoluble αSyn and G85R-SOD1:YFP in the mice resulting from this cross. For this study, we used a slightly different fractionation protocol (see Methods) that enabled detection both soluble and insoluble αSyn in the fractionated lysates of these mice (Additional file 10: Figure S10a and b). By contrast, and in agreement with the histological findings, we could only detect G85R-SOD1:YFP in the soluble fraction from the double transgenic mice (Additional file 10: Figure S10c and d). Collectively, these data indicate that despite robust αSyn pathology and aggregation in spinal cord, the G85R-SOD1:YFP protein remains soluble.

## Discussion

In the current study, we crossed mouse models of tau and αSyn pathologies to mice that express a mutant form of SOD1 that we hypothesized could be vulnerable to proteostasis stress (in this case, the G85R-SOD1:YFP) to provide a reporter of secondary aggregation. Our study is the first that we are aware of to use a fluorescent reporter (e.g., a protein tagged to YFP) to investigate bystander misfolding in a mammalian system in vivo. We observed robust induction of G85R-SOD1:YFP inclusion pathology throughout the neuropil only when the protein was expressed in the JNPL3 model of spinal tauopathy. Importantly, with this model we demonstrate that G85R-SOD1:YFP pathology did not significantly co-localize with tau pathology in the spinal cord of JNPL3-G85R-SOD1:YFP animals, providing evidence against simple co-aggregation of the two proteins. Although it is possible that misfolded mutant tau cross-seeded mutant SOD1 aggregation when co-expressed, we have previously reported that we could not cross-seed G85R-SOD1:YFP aggregation by injecting the spinal cords of these mice with spinal homogenates of paralyzed JNPL3 mice containing pathological tau aggregates [45]. Despite robust burdens of neurodegenerative pathology, we did not observe induced aggregation of G85R-SOD1:YFP in crosses to the spinal model of α-synucleinopathy or the cortical model of tauopathy. Our results suggest a model in which there are cell-type differences in the vulnerability of proteins to bystander misfolding, and a degree of specificity in terms of which proteins succumb to bystander misfold in response to the primary protein pathology.

Evidence of secondary protein misfolding in vivo is relatively scarce. Reports in *C. elegans* were some of the first described instances of secondary misfolding; these made use of temperature-sensitive mutants of paramyosin, dynamin, and ras that failed to achieve active conformations in the presence of aggregation-prone proteins (huntingtin and SOD1) [28, 29]. This finding was originally thought to occur because the expression of the aggregation-prone protein overwhelmed the cellular protein folding machinery, thus leaving TS mutant proteins with too little support to fold correctly. We argue that the G85R-SOD1:YFP construct that is expressed at low levels in the transgenic model used here is a reasonable parallel to the study in *C. elegans* with TS mutant protein. Although we cannot assert that the mutant SOD1 protein is ever completely natively folded, in hemizygous mice the mutant SOD1 protein displays a diffuse cytoplasmic distribution and these mice do not develop evidence of motor neuron disease or the accompanying pathology (astrogliosis or microglosis [43]). However it is clear that the G85R-SOD1:YFP protein is vulnerable to the misfolding associated with aggregation, because it can easily be induced to do so by injecting small amounts of tissues containing aggregates of mutant SOD1 [43–45] or by raising the levels of the protein by generating homozygous mice as was described in the original model [46]. We have also previously observed induction of G85R-SOD1:YFP aggregation in bigenic mice generated by crosses to a transgenic G93A SOD1 animal [43]. Notably, the location and appearance of the fluorescent inclusion pathology we have observed here in bigenic JNPL3/G85R-SOD1:YFP animals is very similar to what we observed in bigenic G93A/G85R-SOD1:YFP mice and G85R-SOD1:YFP mice injected with tissue homogenates containing G93A SOD1 aggregates [43]. Importantly, intraspinal injection of tissue homogenates from paralyzed JNPL3 mice does not induce aggregation of G85R-SOD1:YFP [45]. Thus, in the case of the JNPL3 cross to the G85R-SOD1:YFP mice, we argue that the induced aggregation of the fusion protein is not likely to be due to direct cross seeding, but to an alternative mechanism.

One of the goals of using the G85R-SOD1:YFP mice as a reporter was to identify which cell types were experiencing proteostatic stress to induce secondary misfolding associated with aggregation. Our analysis of the JNPL3 cross to G85R-SOD1:YFP mice revealed the presence of inclusions in the cell bodies of a subset of large motor neurons. However, most of the inclusions were in the neuropil where it is very difficult to ascertain which cell type contains the inclusion. Additionally, as mentioned above, in the interim between when the studies were first initiated and the present we learned that some form of misfolded G85R-SOD1:YFP has the potential to propagate throughout the central nervous system [44]. Thus, it is difficult to decisively conclude whether a cell with a fluorescent inclusion was originally under proteostatic stress or if the inclusion was generated by intracellular spread of aggregate-inducing conformers of G85R-SOD1:YFP originating elsewhere in the CNS [44]. Moreover, the ability of some form of misfolded G85R-SOD1:YFP to mediate cell-to-cell propagation, coupled with the fact that raising expression levels of the protein can induce it to aggregate, makes it very difficult to rule out a scenario in which the presence of tau pathology caused a focal change in expression at some location that created “seeds” of aggregation-prone SOD1 that propagated throughout the spinal axis. Recent transcriptomic studies of mouse models of tauopathy, including the JNPL3 and rTg4510, and transcriptomic studies of human tauopathies (Alzheimer’s Disease [AD] and progressive supranuclear palsy [PSP]), however, have demonstrated that SOD1 expression is not induced in any of these disease settings (Additional file 11; Table S1). The transgene construct used to produce the G85R-SOD1:YFP animals is an engineered fragment of human genomic DNA, which would be expected to be regulated as endogenous SOD1 in humans. Thus, there is little evidence to suspect that the induced misfolding of G85R-SOD1:YFP in the bigenic crosses with JNPL3 mice is due to an induction of the SOD1 transgene expression. The available data suggest that the more likely scenario is that loss of proteostatic function within the spinal axis of the JNPL3/G85R-SOD1:YFP mice led to the induced misfolding and aggregation of the mutant SOD1 reporter.

Our findings in the crosses of G85R-SOD1:YFP mice to JNPL3 mice were in contrast to findings in crosses with mice that develop cortical tau pathology (rTg4510 mice). These findings also contrast to previous observations of what appears to be “secondary induction” of cytoplasmic TDP-43 pathology (a protein that has been pathologically associated with both ALS and FTD) in response to tauopathy in both the JNPL3 and rTg4510 mouse models [35, 57]. In these models, tau pathology usually preceded TDP-43 pathology, suggesting that TDP-43 pathology may largely occur due to the presence of the pathological tau. The lack of G85R-SOD1:YFP inclusion formation in the rTg4510 cross is also remarkable given the fact that SOD1 aggregation is induced by relatively low levels of tau expression in JNPL3 mice (2X endogenous [47]); whereas, the rTg4510 mice produce much higher levels of mutant tau (13X endogenous [49, 50]). Notably, cortical pathology is generally not seen in mice that express mutant SOD1 including the homozygous G85R-SOD1:YFP mice [46]. The expression level of the G85R-SOD1:YFP protein in forebrain is about 2-fold lower than in spinal cord (Additional file 12: Figure S11), which could partially explain the lack of induced mutant SOD1 aggregation in the cross with the rTg4510 mice. However, the rTg4510 mice exhibit profound neurodegenerative changes by 5.5 months of age [50], and it is difficult to accept that the levels of G85R-SOD1:YFP are the sole determinate of whether or not it misfolds and aggregates. Alternatively, it is possible that cortical neurons express unique proteostatic factors that effectively limit the coalescence of misfolded mutant SOD1 into inclusions even in the setting of severe proteostatic distress (see hypothetical model in Additional file 13: Figure S12). Alternatively, there may be differences in the expression levels of one or more proteostatic factors (chaperones, ubiquitin ligases, etc.) between forebrain and spinal cord, such that spinal cord is more vulnerable (Additional file 13: Figure S12). One such factor that has previously been identified by Israelson and colleagues as potentially being responsible for tissue specificity of SOD1 misfolding is macrophage migration inhibitory factor (MIF) [58]. Cells that exhibited MIF localization to the cell bodies were protected from mutant SOD1 aggregation. However, the commercially available antibodies used to study MIF by Israelson in the foregoing study have been discontinued, and antibodies we obtained from other sources did not produce the same staining pattern. Therefore, we were not able to determine the levels or subcellular distribution of MIF in the cortex of the rTg4510 mice might explain our findings. Additionally, it has been shown that spinal motor neurons exhibit a higher threshold for the induction of the protective heat-shock response, specifically regarding a hindered ability in the activation of the transcription factor HSF1 [59]. HSF1 activation leads to the upregulation of the chaperones Hsp70 and Hsp90 which, when induced in mice expressing the G93A mutant SOD1 variant, leads to slower progression of disease [60]. This naturally lower threshold for the heat-shock response could contribute to a higher propensity for mutant SOD1 aggregation in the spinal cord compared to other regions.

Our results from the cross of M83 mice to G85R-SOD1:YFP mice strongly contrasted to the results from the cross with the JNPL3 model. Despite both the JNPL3 and M83 exhibiting robust spinal cord and brainstem pathology, the impact of tau versus αSyn pathology upon our G85R-SOD1:YFP aggregation was dramatically different. This was especially interesting given recent reports suggesting that αSyn and SOD1 interact, leading to increased SOD1 oligomerization [36, 61]. We postulate that our findings reveal the differential effects that tau and αSyn have upon proteostasis and the maintenance of protein folding. More specifically, the proteostatic factors (chaperones, ubiquitin ligases, deubiquitinating enzymes, etc.) that maintain the folding state of tau, αSyn and SOD1 could differ in a manner that leaves mutant SOD1 vulnerable to aggregation in the presence of severe tau pathology, but not in the presence of αSyn pathology (Additional file 13: Figure S12).

Unfortunately, the chaperone subnetworks that are critical in preventing mutant SOD1 aggregation or that are engaged by tau and αSyn pathology remain unclear. Mutant SOD1 has been shown to associate with Hsc70, Hsp70, Hsp27, Hsp25, αB-crystallin and Hsp110 subfamily chaperones [46, 62, 63]. Hsp70 has been shown to bind to the microtubule binding repeats of tau [64], regulating and stabilizing its association with microtubules [65, 66]. Complexes containing the carboxy-terminal Hsp70-interacting protein (CHIP) and either Hsp70 or Hsp90 have been found to promote tau degradation [67–69]. The interactions of tau with small Hsps are slightly less clear; however, Hsp27 has been shown to interact with hyperphosphorylated tau in human AD brain tissue [70]. Hsp70/CHIP have also been found to contribute to αSyn maintenance and combat fibril formation [71–75]. Additionally, the small Hsps (αB-crystallin, Hsp27, Hsp20, HspB8, and HspB2B3) all appear to interact with αSyn (both wild-type and mutant), working to prevent the development of fibrils [76]. Finally, Hsp90 is known to modulate αSyn aggregation, binding to oligomeric species to increase their stability and attenuate toxicity [77, 78]. Deciphering whether there are specific chaperones, or other proteostatic factors, that become engaged in attempting to mitigate misfolded tau in spinal neurons, leaving mutant SOD1 with inadequate access to factors that mitigate its aggregation and will require further study to confirm.

## Conclusions

In conclusion, we have used the co-expression of G85R-SOD1:YFP, a protein that is inherently prone to misfolding, to visualize and investigate “secondary misfolding” in settings of tau and αSyn pathology. The induced aggregation of G85R-SOD1:YFP in the presence of robust spinal tauopathy (as seen in the JNPL3 model) is an outcome consistent with bystander aggregation caused by proteostatic stress. Unexpectedly, the presence of either robust tau pathology in cortical neurons or αSyn pathology in the spinal cord, which in both cases causes severe degenerative changes, did not induce G85R-SOD1:YFP aggregation. One hypothesis that could explain this outcome is that tau and SOD1 have overlapping demands for proteostatic factors that are in limited supply in the spinal axis, such that the presence of the misfolded tau essentially competes for a factor, or factors, that are critical in preventing the aggregation of mutant SOD1 in spinal cord (Additional file 13: Figure S12). Further studies will be required to understand the molecular basis for selective induction of G85R-SOD1:YFP aggregation in these models. That being said, the G85R-SOD1:YFP model has nonetheless proved useful in clearly demonstrating that induced misfolding of one protein by another in neurodegenerative conditions is more complex than simple proteostatic failure. The complexity of bystander misfolding in response to proteinopathies could provide a basis for distinctive clinical symptoms associated with these disorders.

## Additional files

Additional file 1: **Figure S1.** Kaplan-Meier survival curves in JNPL3/G85R-SOD1:YFP mice relative to single transgenic JNPL3 controls. JNPL3 mice expressing the G85R-SOD1:YFP reporter protein (*n* = 8) that exhibited SOD1 inclusion pathology reached an end-stage phenotype significantly faster than JNPL3 transgenic mice (*n* = 7) (*p* < 0.05, Mantel-Cox test). The graphed data originated from six bigenic JNPL3/G85R-SOD1:YFP mice and seven tau-only transgenic JNPL3 mice that were littermate controls. All mice were female. Figure generated using GraphPad Prism (version 7.0 h). (TIFF 90 kb)

Additional file 2: **Figure S2.** Low power views of G85R-SOD1:YFP pathology in the spinal cord of bigenic JNPL3-G85R-SOD1:YFP mice **(a)**. The box marks the position of the image shown in Fig. 1d of the main text. Low power view of fluorescence in mice expressing G85R-SOD1:YFP alone **(c)**. Images shows midsagittal brain section **(b)**. Nuclei were stained with DAPI (blue). The left and right arrows are drawn to magnified regions that are shown in the top left and top right of **(b)**, respectively. Images shown are representative of 8 JNPL3-G85R-SOD1:YFP mice and 3 G85R-SOD1:YFP mice. (TIF 8095 kb)

Additional file 3: **Figure S3.** Primary pathology burden in the JNPL3 spinal cord relative to those crossed to G85R-SOD1:YFP mice. JNPL3 mice **(a)** and those on the G85R-SOD1:YFP background **(b)** were stained with the MC1 antibody (misfolded human tau). Lumbar spinal cord sections are shown. Scale bar; 900 μm. (TIF 1483 kb)

Additional file 4: **Figure S4.** Solubility of SOD1 in JNPL3/G85R-SOD1:YFP mice. For these immunoblots, we used a previously described method of detergent extraction and sedimentation (see Methods). To observe aberrant disulfide cross-links that form as mutant SOD1 aggregates, we performed the SDS-PAGE in the absence of reducing agent (**a** and **b**). In JNPL3/G85R-SOD1:YFP mice versus G85R-SOD1:YFP mice, soluble G85R-SOD1:YFP exists predominantly in higher molecular weight states in double transgenic mice versus single transgenic controls (**a**). However, aggregates of G85R-SOD1:YFP were detected in NP40-insoluble fractions only in the bigenic, paralyzed, mice (**b**). 20 μg protein loaded for all samples. Mouse/human SOD1 was detected using an in-house generated antibody. *n* = 3 per genotype. (TIF 293 kb)

Additional file 5: **Figure S5.** Localization of phosphotau immunoreactivity versus G85R-SOD1:YFP pathology in bigenic JNPL3-G85R-SOD1:YFP mice. Hyperphosphorylated human tau pathology recognized by the AT8 (Ser202/Thr205) antibody appears similar in JNPL3 single transgenic versus JNPL3- 85R-SOD1:YFP bigenic mice **(a, b)**. G85R-SOD1:YFP pathology, detected by an YFP antibody **(c)**, does not robustly co-localize with tau pathology in double transgenic mice **(d)**. Nuclei were stained with DAPI (blue). Images are of 60× magnification within the ventral horn of the spinal cord of JNPL3 and JNPL3-G85R-SOD1:YFP mice. (TIF 869 kb)

Additional file 6: **Figure S6.** Primary pathology burden in the rTg4510 transgenic mouse cortex relative to those crossed to the G85R-SOD1:YFP mouse. rTg4510 mice **(a)** compared to trigenic rTg4510**/**G85R-SOD1:YFP mice (b) after immunostaining with the MC1 antibody (misfolded human tau). Scale bar; 300 μm. (TIF 2770 kb)

Additional file 7: **Figure S7.** Quantification of G85R-SOD1:YFP levels between G85R-SOD1:YFP and rTg4510/G85R-SOD1:YFP mice using direct fluorescence and immunoblot densitometric analysis. Quantification of fluorescence intensity reveals a significantly more intense YFP fluorescence in rTg4510/G85R-SOD1:YFP mice (abbreviated rTg4510-SOD1) compared to G85R-SOD1:YFP controls (abbreviated SOD1) (*n* = 4) **(a)**. However, immunoblot analysis using an antibody to both mouse and human SOD1 demonstrates no statistical difference between levels of G85R-SOD1:YFP in the two mouse groups **(b, c)** (n = 3 per genotype). Endogenous mouse SOD1 (mSOD1) was used as a loading control, and was detected on the same blot shown. Statistical analysis was conducted using GraphPad Prism (version 7.0 h). Error bars show mean ± S.D.; unpaired, two tailed, T-test revealed a significant difference in fluorescence intensity in forebrain by genotype (*p* < 0.01). n.s.; not significant. (TIF 172 kb)

Additional file 8: **Figure S8.** Primary pathology burden in the M83 transgenic mouse spinal cord relative to M83/G85R-SOD1:YFP mice. M83 only mice **(a)** compared to M83/G85R-SOD1:YFP mice **(b)** after injection with αSyn fibrils to induce αSyn pathology. Sections were stained with the 81A antibody (pSer129 αSyn). Lumbar spinal cord sections are shown. Scale bar; 900 μm. (TIF 2919 kb)

Additional file 9: **Figure S9.** Kaplan-Meier survival curves for M83/G85R-SOD1:YFP mice relative to single transgenic M83 controls. All mice were injected intramuscularly with αSyn fibrils to induce pathology. M83 mice expressing the G85R-SOD1:YFP reporter protein that exhibited SOD1 inclusion pathology did not reach an end-stage phenotype significantly faster than M83 transgenic mice. The graphed data originated from 11 bigenic M83/G85R-SOD1:YFP mice (6 female, 5 male) and 15 single transgenic M83 mice (10 male, 5 female) that were littermate controls. Figure generated using GraphPad Prism (version 7.0 h). (TIFF 87 kb)

Additional file 10: **Figure S10.** Solubility of αSyn and SOD1 in M83/G85R-SOD1:YFP mice. No changes in soluble versus NP40-insoluble αSyn were observed between M83/G85R-SOD1:YFP versus G85R-SOD1:YFP mice (**a** and **b**). For these immunoblots we used a sequential fractionation protocol that produced a PBS-soluble fraction and an NP40-insoluble fractions (see Methods). Here we controlled sample concentration by resuspending the NP40-insoluble fraction in a volume equivalent to the initial PBS soluble fraction. Equivalent amounts of each fraction were analyzed by SDS-PAGE (30 μL per sample). We used antibodies to GAPDH as a loading control in soluble fractions on the same blot (**a**). Soluble G85R-SOD1:YFP and endogenous mouse SOD1 was detected in both animal groups (**c**), and insoluble G85R-SOD1:YFP was not detected in either group (**d**). αSyn was detected using the 94-3A10 antibody (provided by the laboratory of Benoit Giasson [79]), while mouse/human SOD1 was detected using an in-house generated antibody. *n* = 3 per genotype. (TIF 284 kb)

Additional file 11: **Table S1.** RNAseq expression data for SOD1 in mouse and human tauopathies. Transcriptomic data from studies of the rTg4510 and JNPL3 mouse models, and from studies of humans brain tissues from Alzheimer disease (AD) and progressive supranuclear palsy (PSP) cases available in https://www.synapse.org/#!Synapse:syn2580853/wiki/409840. (TIF 694 kb)

Additional file 12: **Figure S11.** G85R-SOD1:YFP expression in spinal cord is 2-fold higher than forebrain in G85R-SOD1:YFP heterozygous mice. Forebrain and spinal cord tissue were extracted and 20 μg of protein was used for immunoblot analysis of SOD1 levels, using an antibody specific for human SOD1 (hSOD1) **(a)**. Each lane represents an individual animal (n = 3). Graph represents densitometric quantification of hSOD1 levels normalized to GAPDH **(b)**. Statistical analysis was conducted using GraphPad Prism (version 7.0 h) Error bars show mean ± S.D.; unpaired T-test. A.U.; arbitrary units. (TIF 248 kb)

Additional file 13: **Figure S12.** Hypothetical mechanism of differential effects of tauopathy versus synucleinopathy on G85R-SOD1:YFP secondary aggregation in the spinal cord and cortex. In the JNPL3 spinal cord, misfolded tau occupies proteostatic factors (Factor X) that the mutant SOD1 reporter is also dependent upon for folding or degradation. In the cortex of rTg4510 mice, the levels of Factor X could be higher, or other proteostatic factors specific to brain (Factor Y) could be present to prevent the aggregation mutant SOD1. Meanwhile, misfolded αSyn occupies proteostatic factors distinct from those of tau (Factor Z), leaving a sufficient level of Factor X to prevent the aggregation of mutant SOD1. (TIF 553 kb)

## Abbreviations

AD: Alzheimer’s disease
ALS: Amyotrophic lateral sclerosis
CaMKII: Calmodulin kinase II
CHIP: Carboxy-terminal Hsp70-interacting protein
CNS: Central nervous system
FTD: Frontotemporal dementia
G85R-SOD1:YFP: Superoxide dismutase 1 tagged to YFP containing the G85R mutation
GFP: Green fluorescent protein
IACUC: Institutional animal care and use committee
MIF: Macrophage migration inhibitory factor
PD: Parkinson’s disease
polyQ: Polyglutamine
TS: Temperature-sensitive
tTA: Tet-transactivator
UPS: Ubiquitin-proteasome system
αSyn: α-synuclein
