# Supplementary figures and images for: Differential induction of mutant SOD1 misfolding and aggregation by tau and α-synuclein pathology
Source: Mol Neurodegener. 2018 May 18;13:23. doi: 10.1186/s13024-018-0253-9 (PMC5960184; doi:10.1186/s13024-018-0253-9)

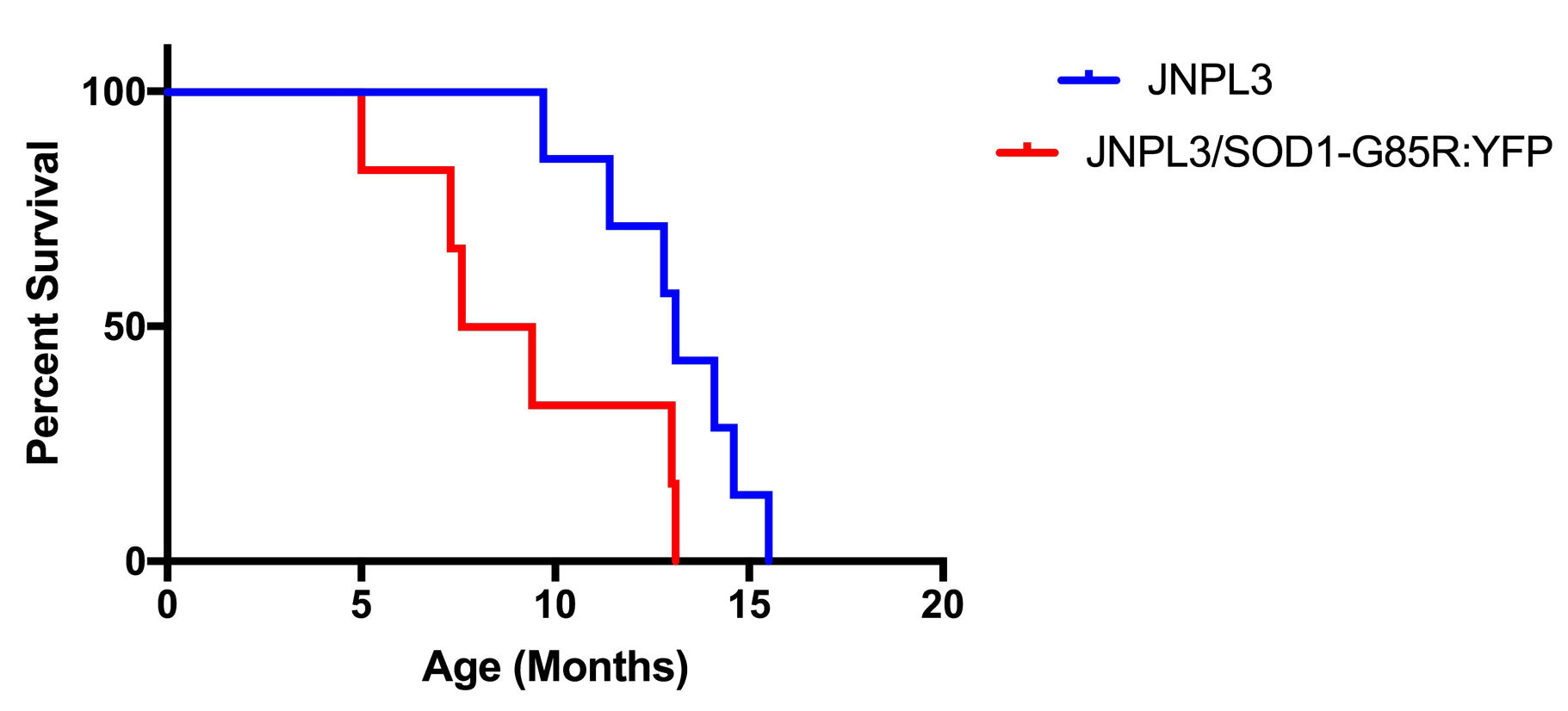

Supplement: Supplementary file 1 — Figure S1. Kaplan-Meier survival curves in JNPL3/G85R-SOD1:YFP mice relative to single transgenic JNPL3 controls. JNPL3 mice expressing the G85R-SOD1:YFP reporter protein (n = 8) that exhibited SOD1 inclusion pathology reached an end-stage phenotype significantly faster than JNPL3 transgenic mice (n = 7) (p < 0.05, Mantel-Cox test). The graphed data originated from six bigenic JNPL3/G85R-SOD1:YFP mice and seven tau-only transgenic JNPL3 mice that were littermate controls. All mice were female. Figure generated using GraphPad Prism (version 7.0 h). (TIFF 90 kb) [file 13024_2018_253_MOESM1_ESM.tiff]

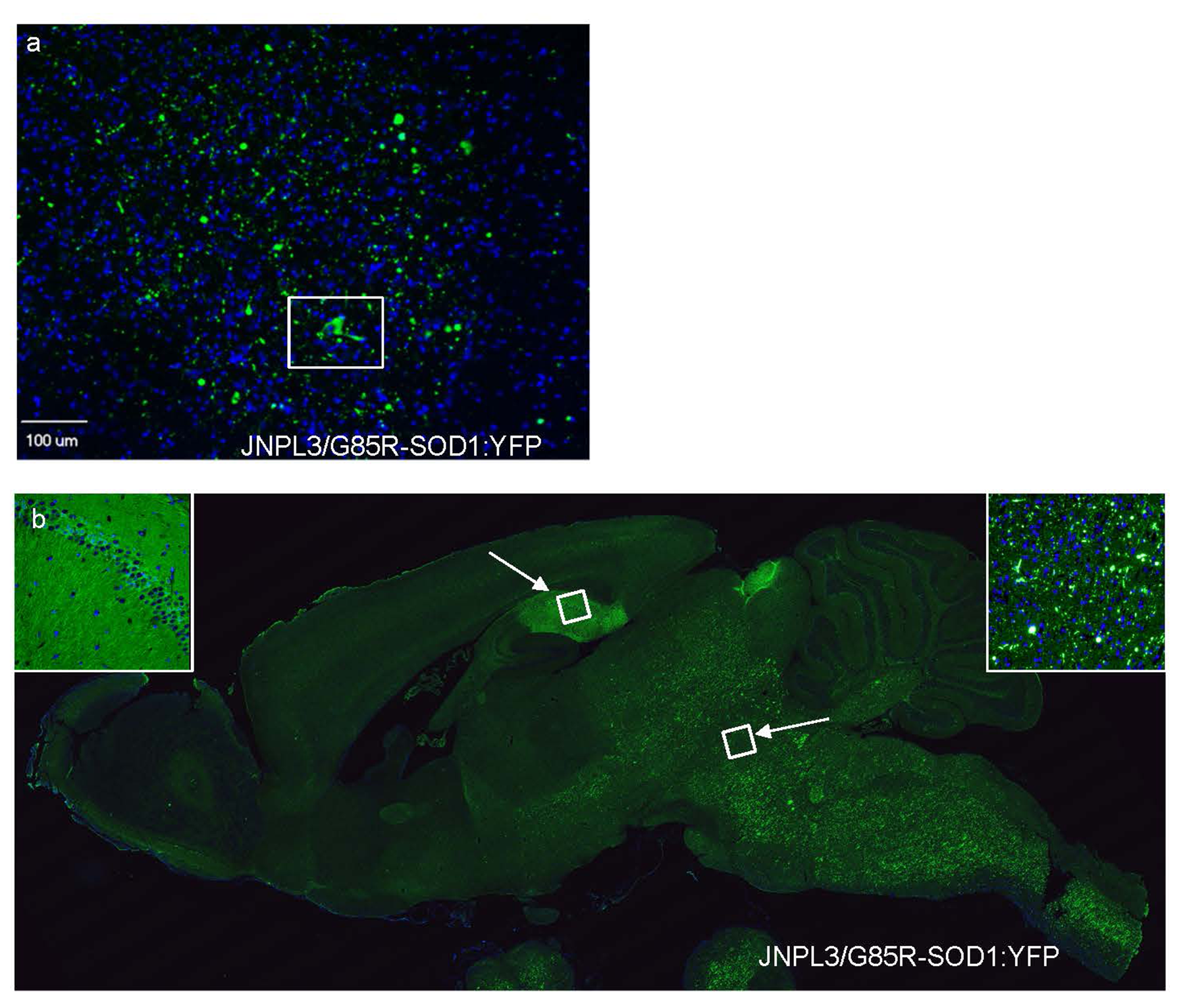

Supplement: Supplementary file 2 — Figure S2. Low power views of G85R-SOD1:YFP pathology in the spinal cord of bigenic JNPL3-G85R-SOD1:YFP mice (a). The box marks the position of the image shown in Fig. 1d of the main text. Low power view of fluorescence in mice expressing G85R-SOD1:YFP alone (c). Images shows midsagittal brain section (b). Nuclei were stained with DAPI (blue). The left and right arrows are drawn to magnified regions that are shown in the top left and top right of (b), respectively. Images shown are representative of 8 JNPL3-G85R-SOD1:YFP mice and 3 G85R-SOD1:YFP mice. (TIF 8095 kb) [file 13024_2018_253_MOESM2_ESM.tif]

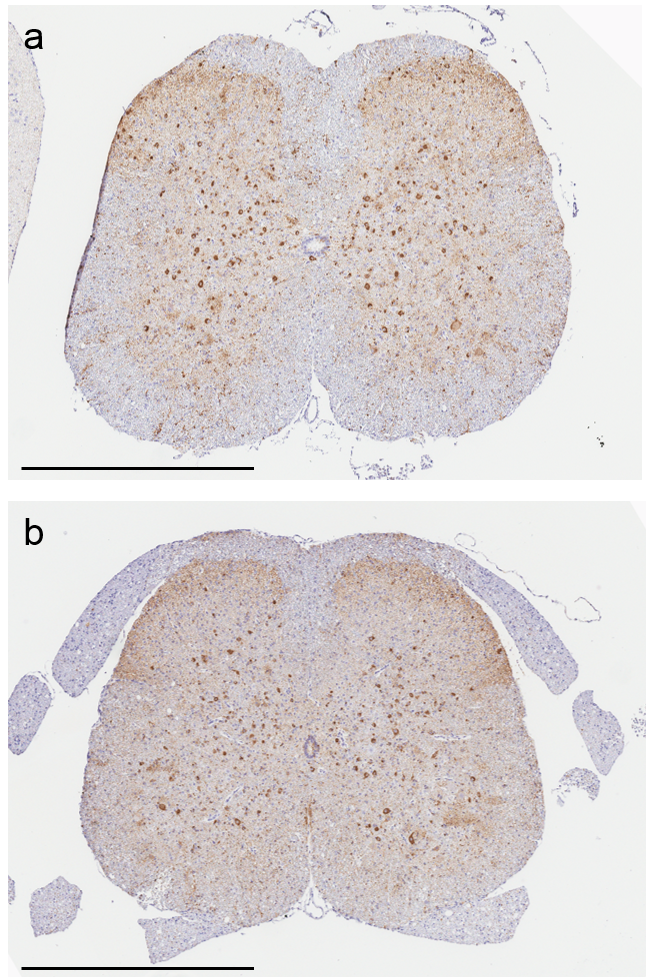

Supplement: Supplementary file 3 — Figure S3. Primary pathology burden in the JNPL3 spinal cord relative to those crossed to G85R-SOD1:YFP mice. JNPL3 mice (a) and those on the G85R-SOD1:YFP background (b) were stained with the MC1 antibody (misfolded human tau). Lumbar spinal cord sections are shown. Scale bar; 900 μm. (TIF 1483 kb) [file 13024_2018_253_MOESM3_ESM.tif]

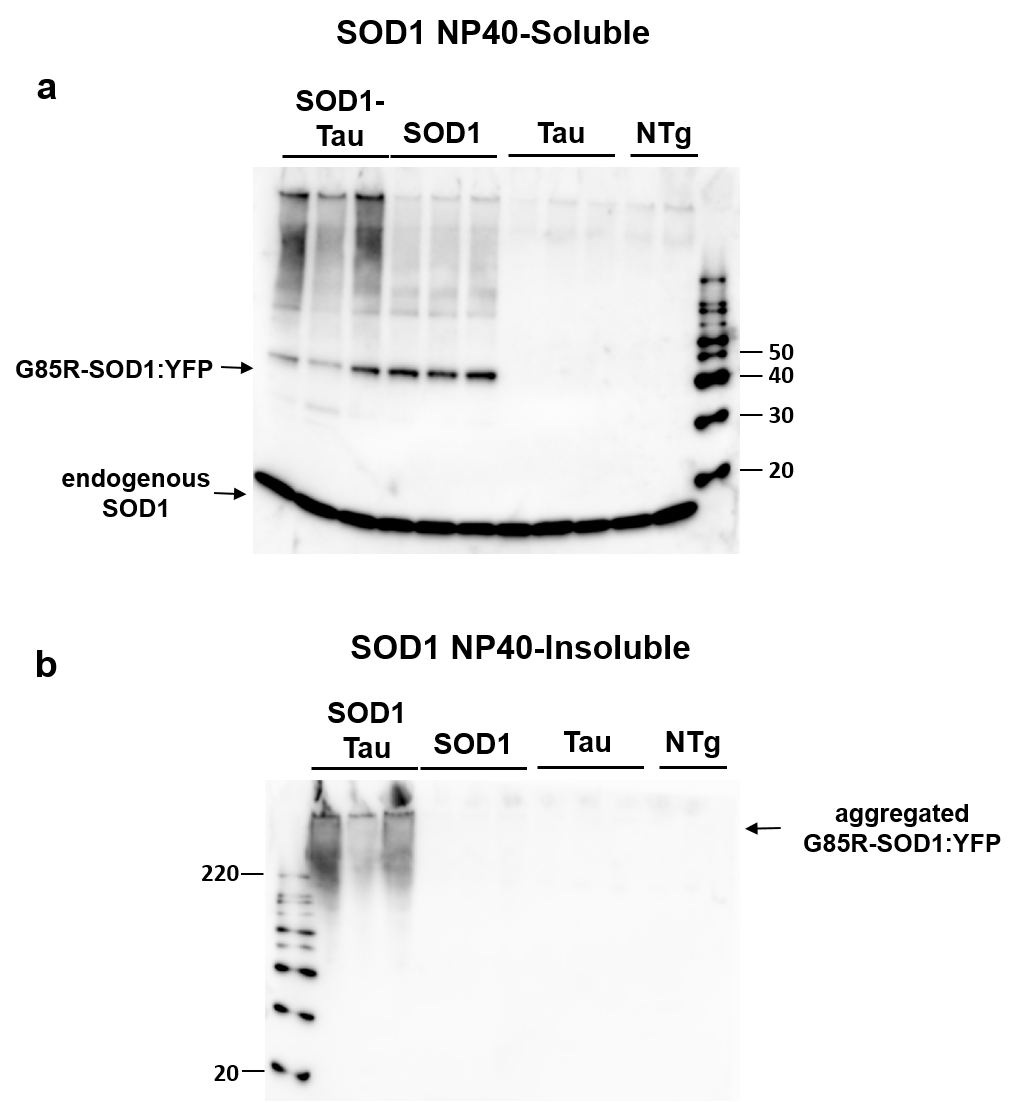

Supplement: Supplementary file 4 — Figure S4. Solubility of SOD1 in JNPL3/G85R-SOD1:YFP mice. For these immunoblots, we used a previously described method of detergent extraction and sedimentation (see Methods). To observe aberrant disulfide cross-links that form as mutant SOD1 aggregates, we performed the SDS-PAGE in the absence of reducing agent (a and b). In JNPL3/G85R-SOD1:YFP mice versus G85R-SOD1:YFP mice, soluble G85R-SOD1:YFP exists predominantly in higher molecular weight states in double transgenic mice versus single transgenic controls (a). However, aggregates of G85R-SOD1:YFP were detected in NP40-insoluble fractions only in the bigenic, paralyzed, mice (b). 20 μg protein loaded for all samples. Mouse/human SOD1 was detected using an in-house generated antibody. n = 3 per genotype. (TIF 293 kb) [file 13024_2018_253_MOESM4_ESM.tif]

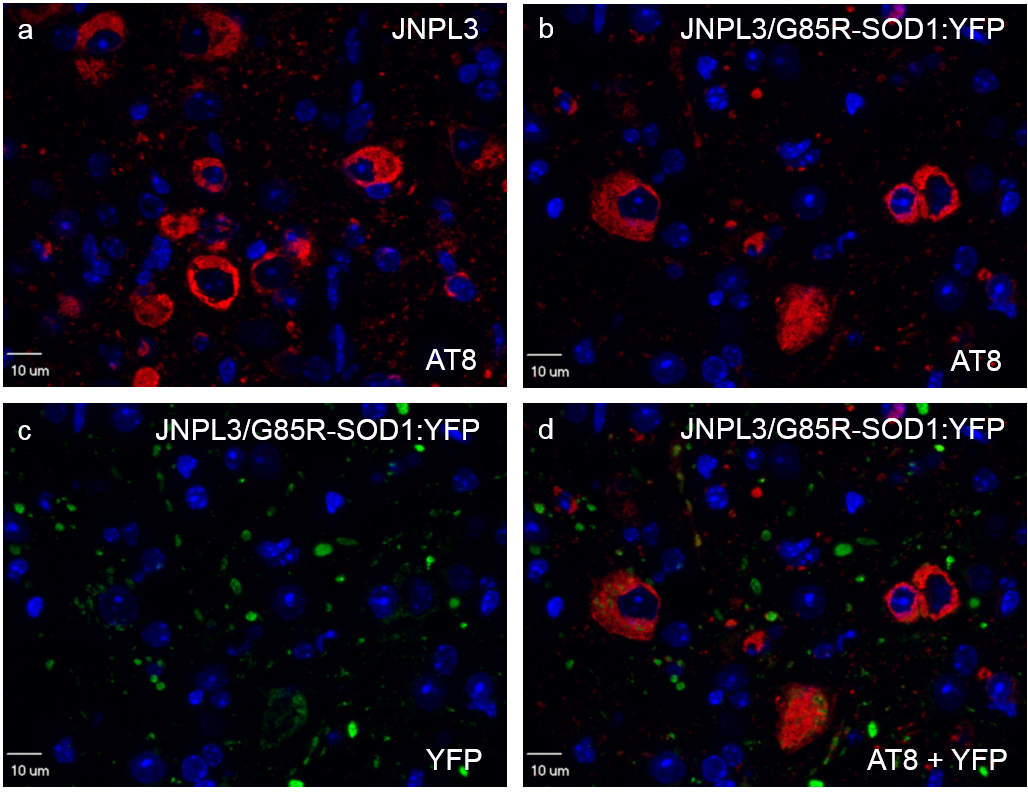

Supplement: Supplementary file 5 — Figure S5. Localization of phosphotau immunoreactivity versus G85R-SOD1:YFP pathology in bigenic JNPL3-G85R-SOD1:YFP mice. Hyperphosphorylated human tau pathology recognized by the AT8 (Ser202/Thr205) antibody appears similar in JNPL3 single transgenic versus JNPL3- 85R-SOD1:YFP bigenic mice (a, b). G85R-SOD1:YFP pathology, detected by an YFP antibody (c), does not robustly co-localize with tau pathology in double transgenic mice (d). Nuclei were stained with DAPI (blue). Images are of 60× magnification within the ventral horn of the spinal cord of JNPL3 and JNPL3-G85R-SOD1:YFP mice. (TIF 869 kb) [file 13024_2018_253_MOESM5_ESM.tif]

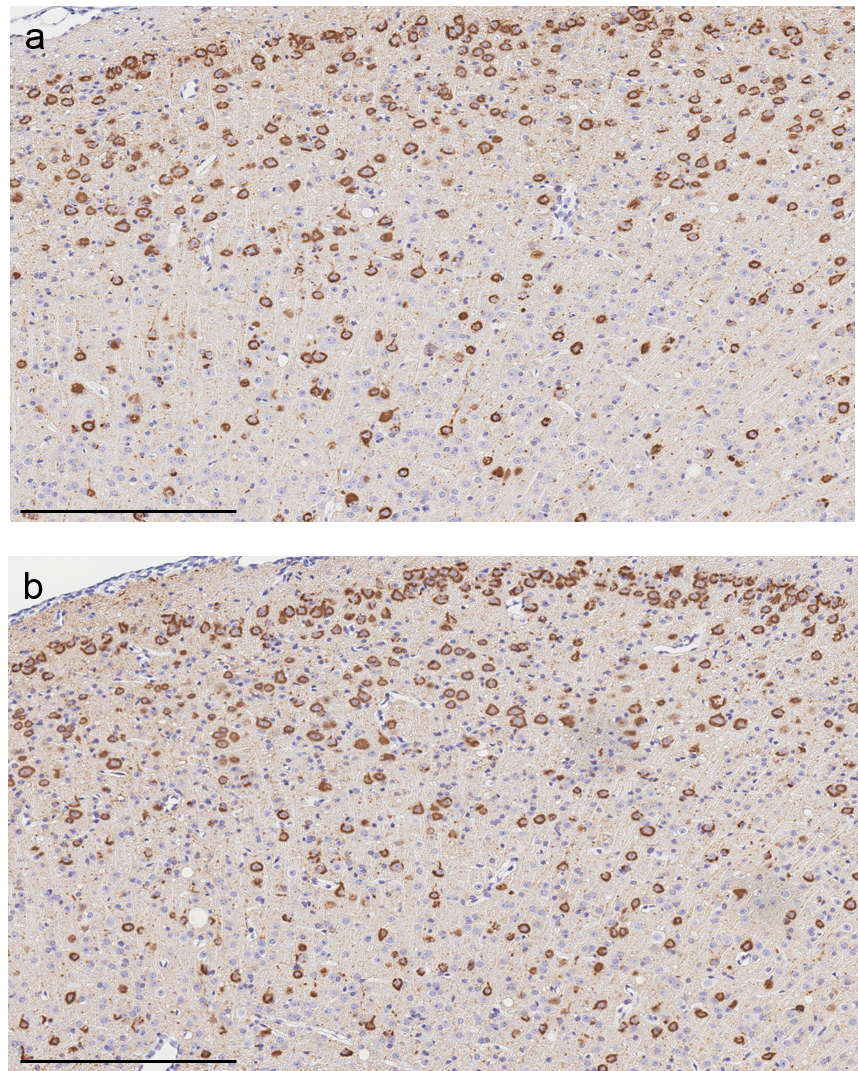

Supplement: Supplementary file 6 — Figure S6. Primary pathology burden in the rTg4510 transgenic mouse cortex relative to those crossed to the G85R-SOD1:YFP mouse. rTg4510 mice (a) compared to trigenic rTg4510/G85R-SOD1:YFP mice (b) after immunostaining with the MC1 antibody (misfolded human tau). Scale bar; 300 μm. (TIF 2770 kb) [file 13024_2018_253_MOESM6_ESM.tif]

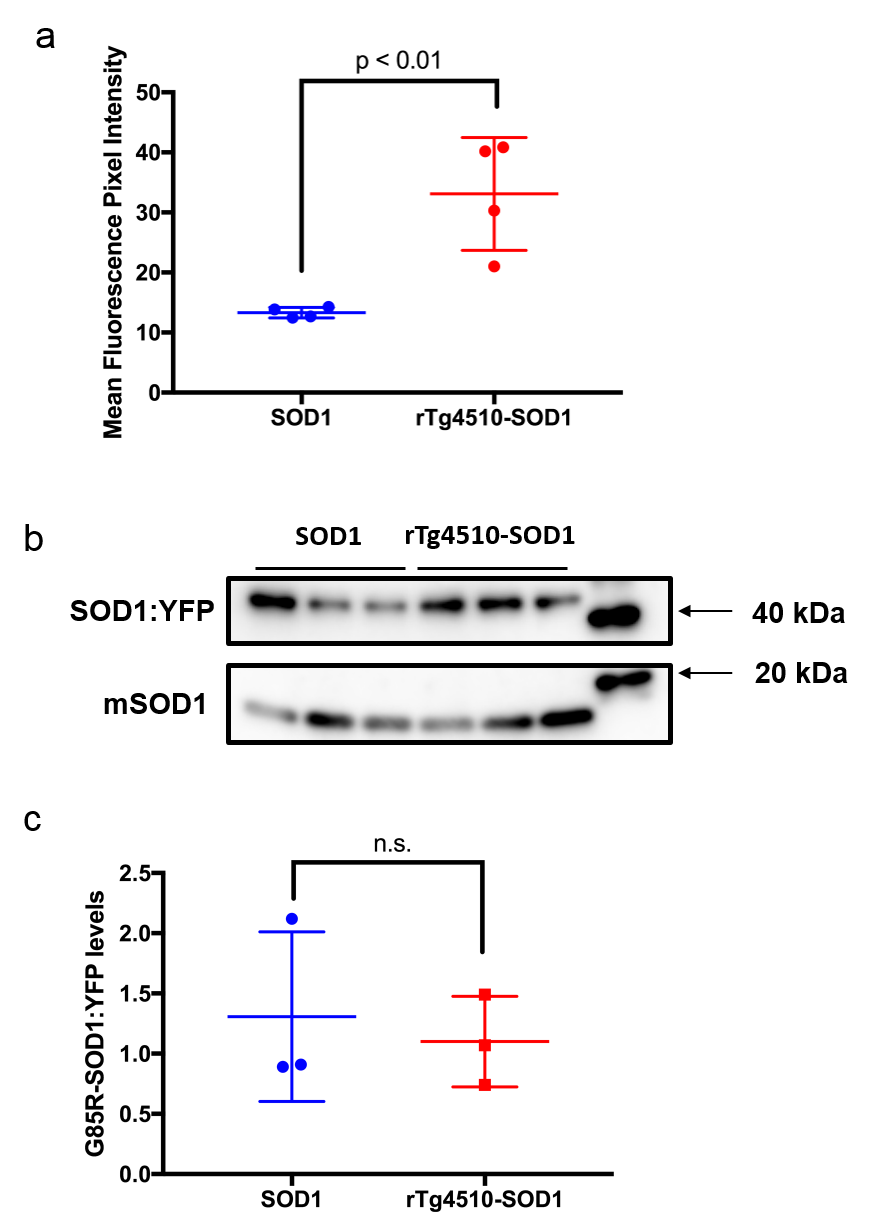

Supplement: Supplementary file 7 — Figure S7. Quantification of G85R-SOD1:YFP levels between G85R-SOD1:YFP and rTg4510/G85R-SOD1:YFP mice using direct fluorescence and immunoblot densitometric analysis. Quantification of fluorescence intensity reveals a significantly more intense YFP fluorescence in rTg4510/G85R-SOD1:YFP mice (abbreviated rTg4510-SOD1) compared to G85R-SOD1:YFP controls (abbreviated SOD1) (n = 4) (a). However, immunoblot analysis using an antibody to both mouse and human SOD1 demonstrates no statistical difference between levels of G85R-SOD1:YFP in the two mouse groups (b, c) (n = 3 per genotype). Endogenous mouse SOD1 (mSOD1) was used as a loading control, and was detected on the same blot shown. Statistical analysis was conducted using GraphPad Prism (version 7.0 h). Error bars show mean ± S.D.; unpaired, two tailed, T-test revealed a significant difference in fluorescence intensity in forebrain by genotype (p < 0.01). n.s.; not significant. (TIF 172 kb) [file 13024_2018_253_MOESM7_ESM.tif]

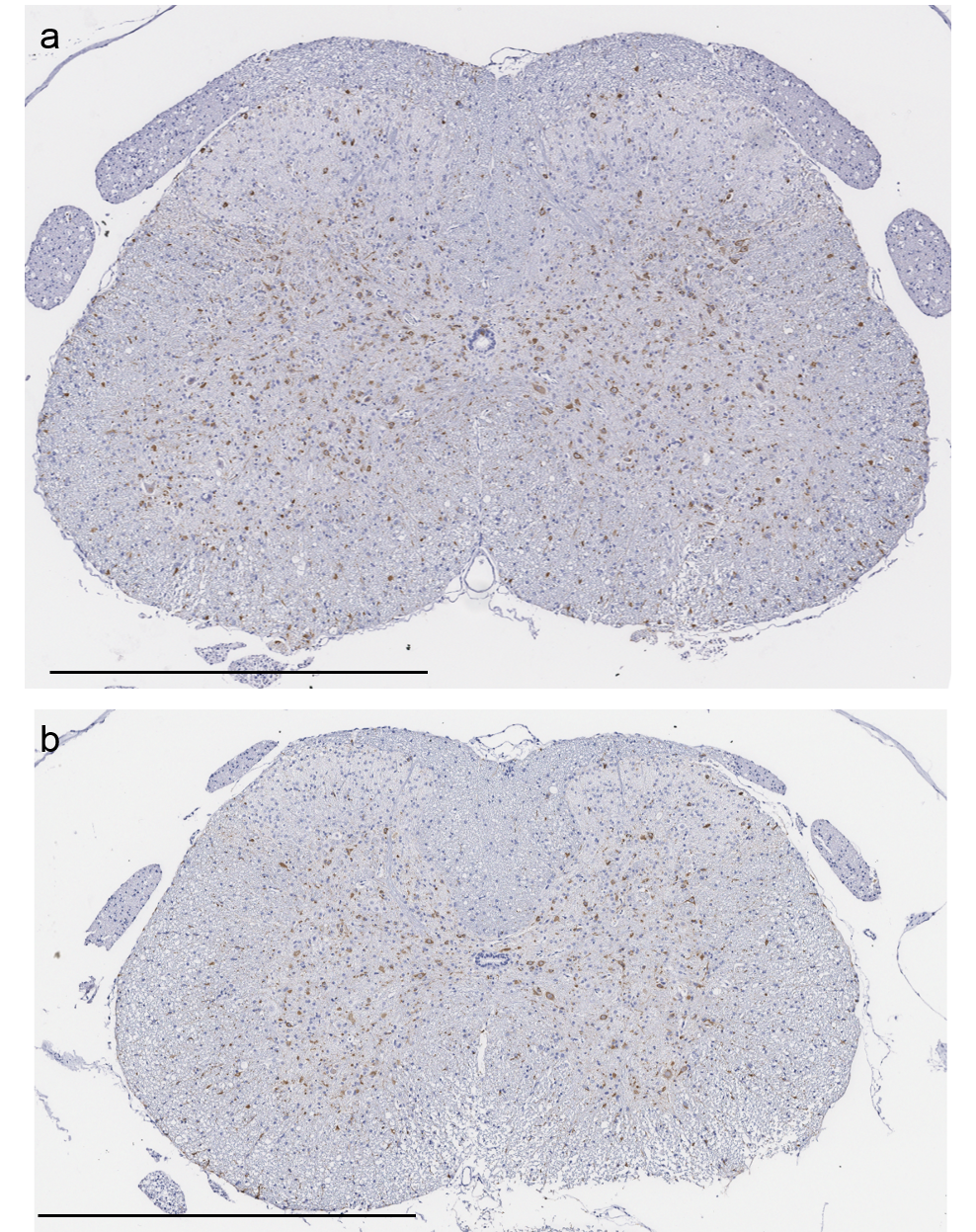

Supplement: Supplementary file 8 — Figure S8. Primary pathology burden in the M83 transgenic mouse spinal cord relative to M83/G85R-SOD1:YFP mice. M83 only mice (a) compared to M83/G85R-SOD1:YFP mice (b) after injection with αSyn fibrils to induce αSyn pathology. Sections were stained with the 81A antibody (pSer129 αSyn). Lumbar spinal cord sections are shown. Scale bar; 900 μm. (TIF 2919 kb) [file 13024_2018_253_MOESM8_ESM.tif]

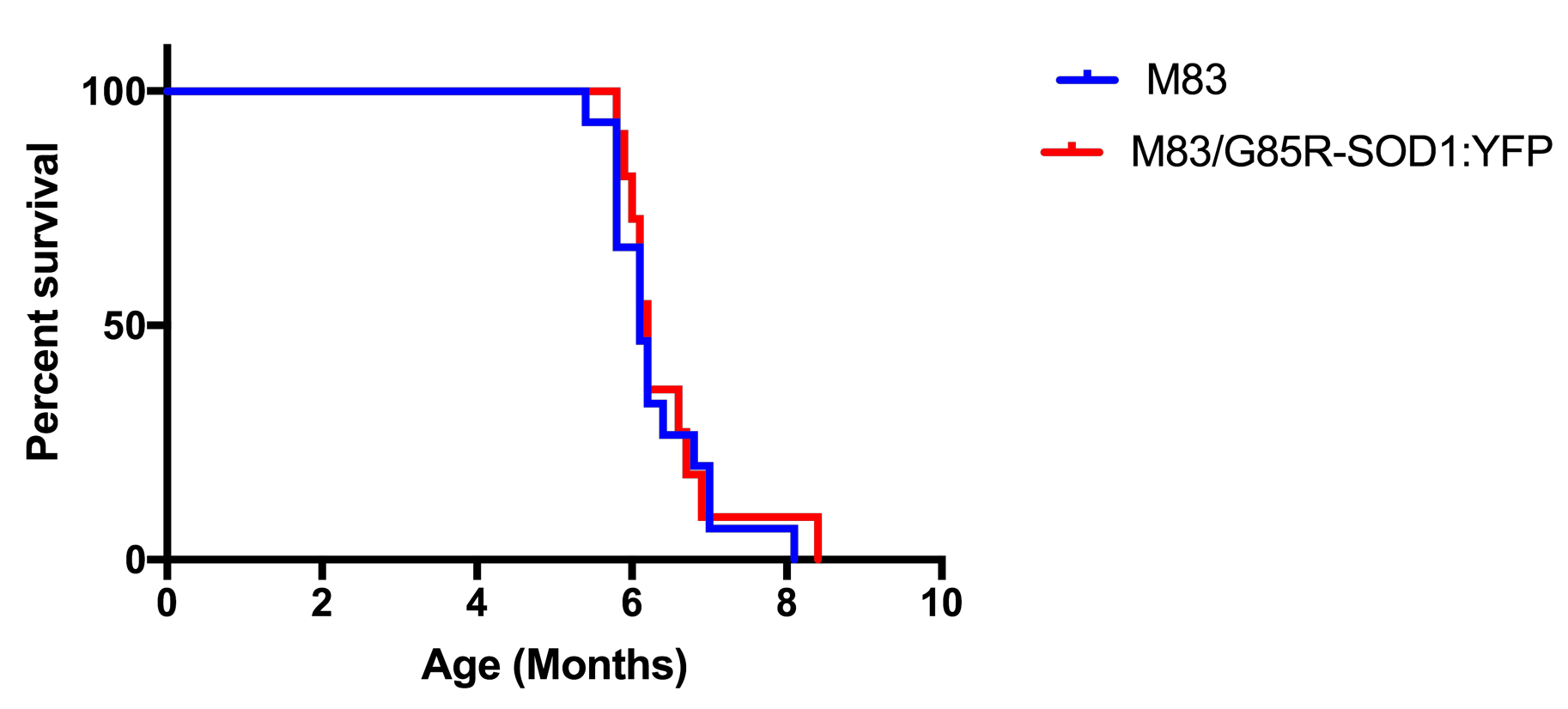

Supplement: Supplementary file 9 — Figure S9. Kaplan-Meier survival curves for M83/G85R-SOD1:YFP mice relative to single transgenic M83 controls. All mice were injected intramuscularly with αSyn fibrils to induce pathology. M83 mice expressing the G85R-SOD1:YFP reporter protein that exhibited SOD1 inclusion pathology did not reach an end-stage phenotype significantly faster than M83 transgenic mice. The graphed data originated from 11 bigenic M83/G85R-SOD1:YFP mice (6 female, 5 male) and 15 single transgenic M83 mice (10 male, 5 female) that were littermate controls. Figure generated using GraphPad Prism (version 7.0 h). (TIFF 87 kb) [file 13024_2018_253_MOESM9_ESM.tiff]

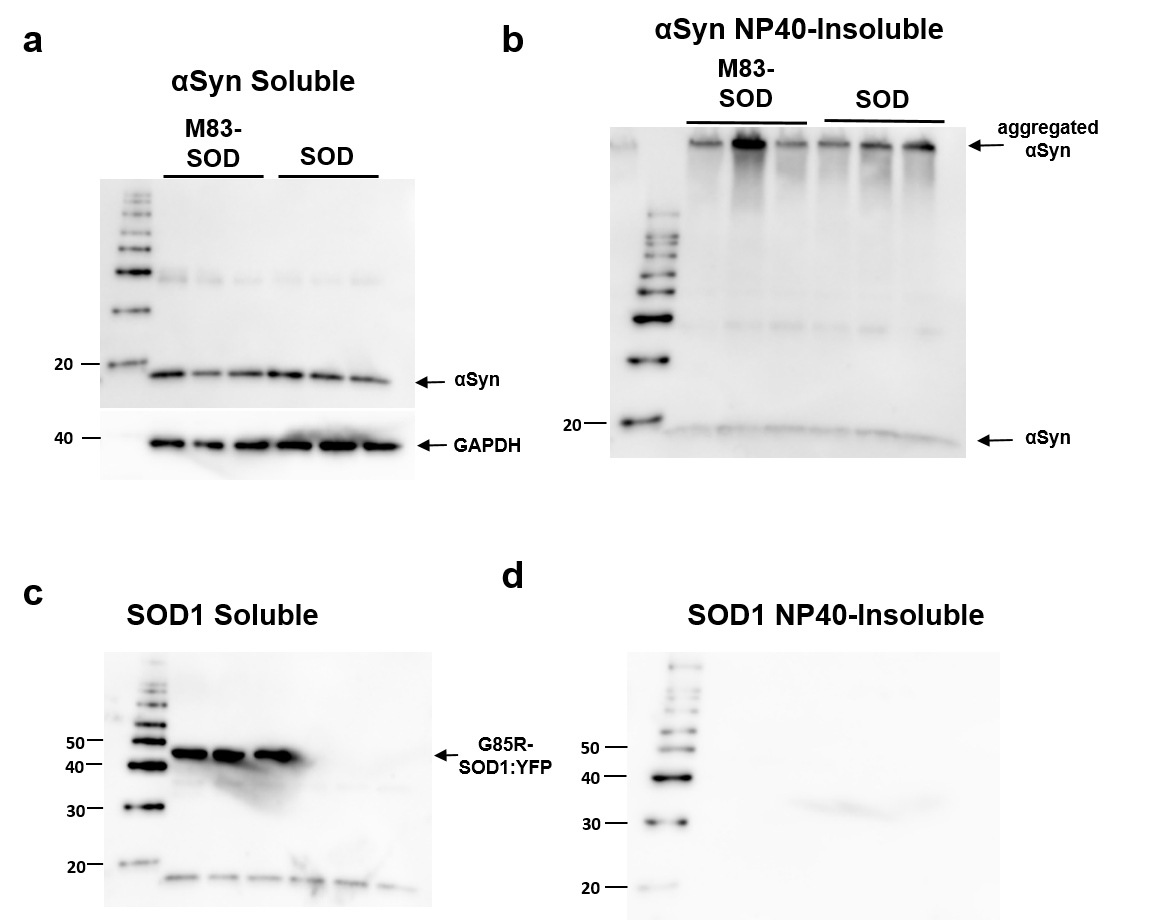

Supplement: Supplementary file 10 — Figure S10. Solubility of αSyn and SOD1 in M83/G85R-SOD1:YFP mice. No changes in soluble versus NP40-insoluble αSyn were observed between M83/G85R-SOD1:YFP versus G85R-SOD1:YFP mice (a and b). For these immunoblots we used a sequential fractionation protocol that produced a PBS-soluble fraction and an NP40-insoluble fractions (see Methods). Here we controlled sample concentration by resuspending the NP40-insoluble fraction in a volume equivalent to the initial PBS soluble fraction. Equivalent amounts of each fraction were analyzed by SDS-PAGE (30 μL per sample). We used antibodies to GAPDH as a loading control in soluble fractions on the same blot (a). Soluble G85R-SOD1:YFP and endogenous mouse SOD1 was detected in both animal groups (c), and insoluble G85R-SOD1:YFP was not detected in either group (d). αSyn was detected using the 94-3A10 antibody (provided by the laboratory of Benoit Giasson [79]), while mouse/human SOD1 was detected using an in-house generated antibody. n = 3 per genotype. (TIF 284 kb) [file 13024_2018_253_MOESM10_ESM.tif]

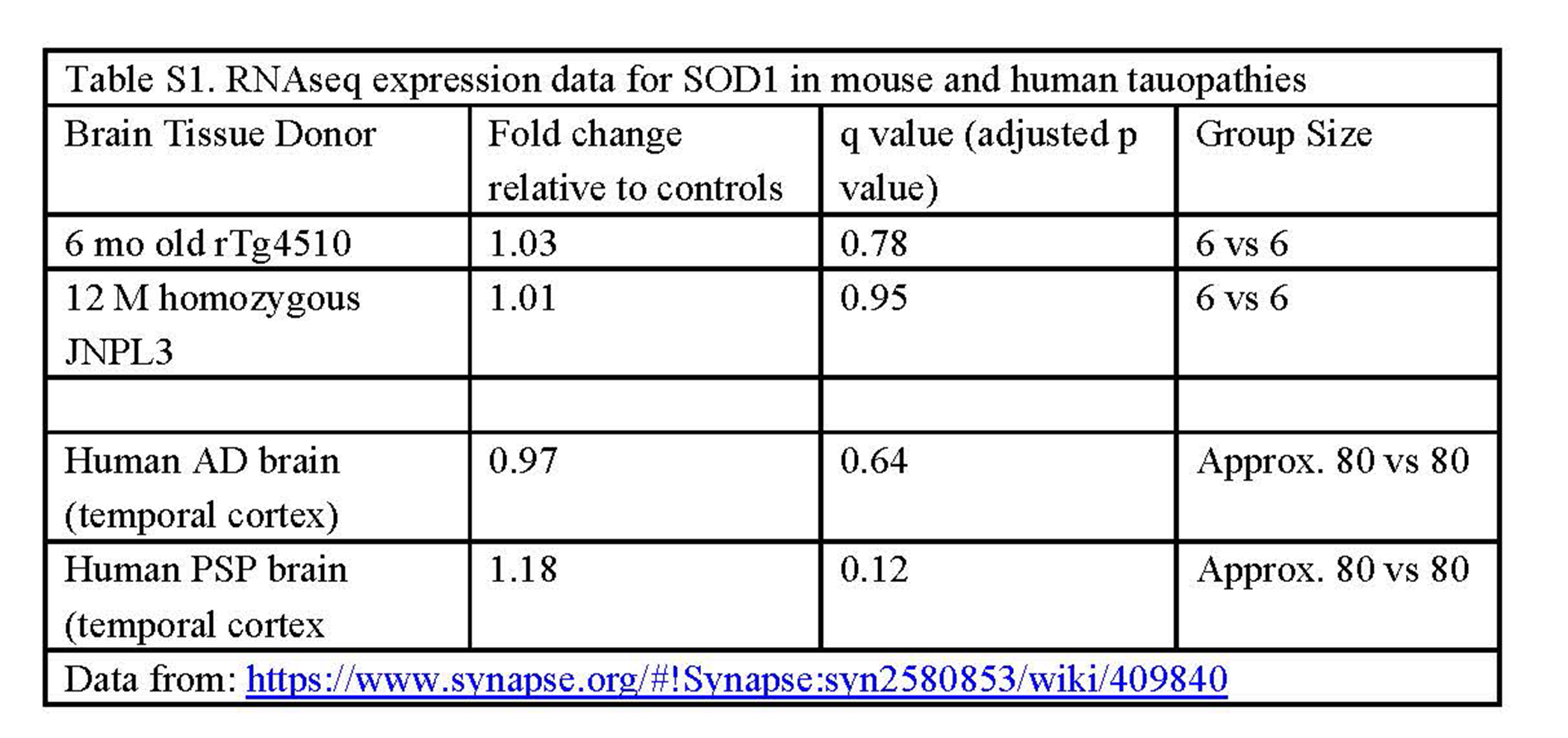

Supplement: Supplementary file 11 — Table S1. RNAseq expression data for SOD1 in mouse and human tauopathies. Transcriptomic data from studies of the rTg4510 and JNPL3 mouse models, and from studies of humans brain tissues from Alzheimer disease (AD) and progressive supranuclear palsy (PSP) cases available in https://www.synapse.org/#!Synapse:syn2580853/wiki/409840. (TIF 694 kb) [file 13024_2018_253_MOESM11_ESM.tif]

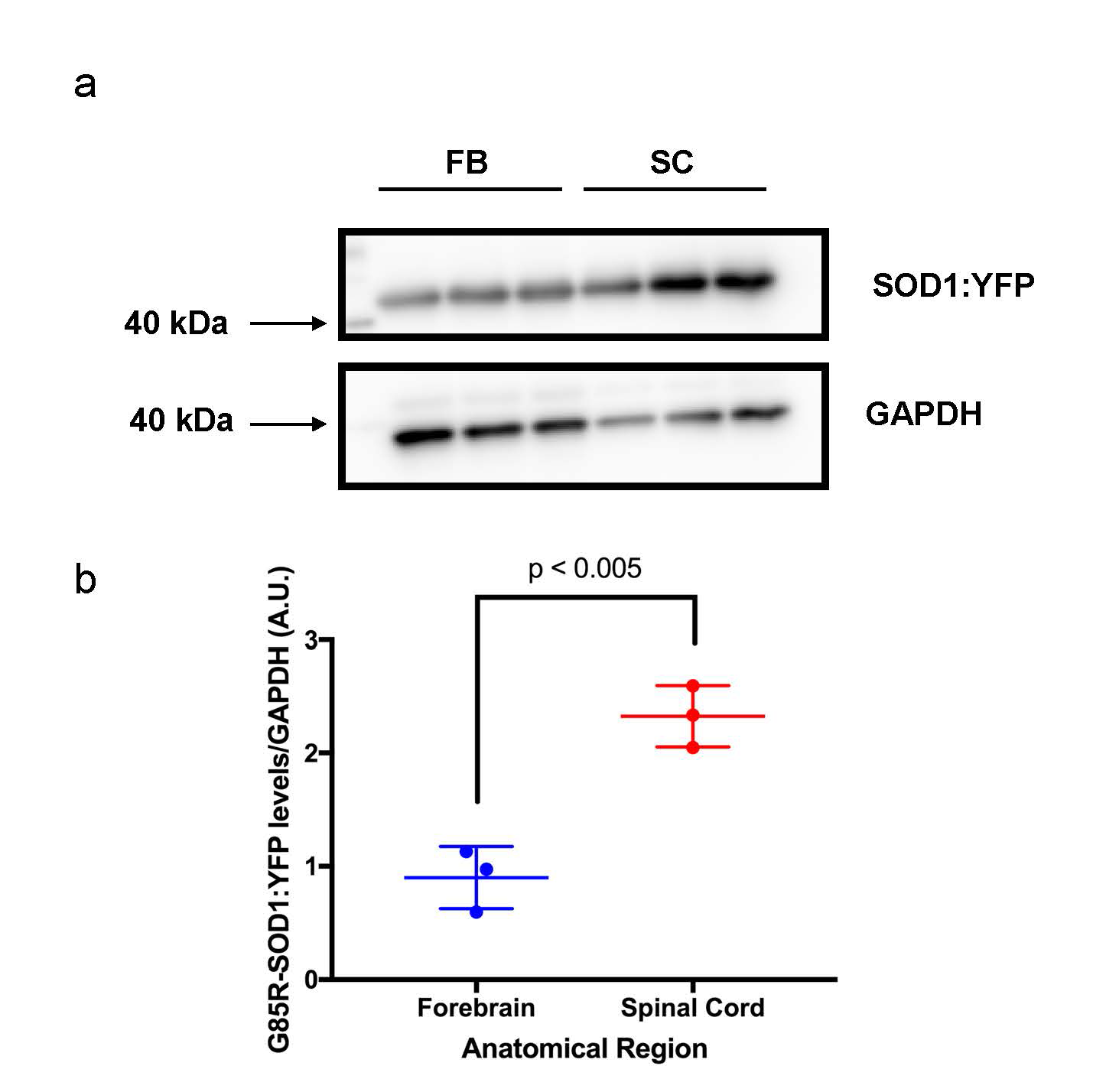

Supplement: Supplementary file 12 — Figure S11. G85R-SOD1:YFP expression in spinal cord is 2-fold higher than forebrain in G85R-SOD1:YFP heterozygous mice. Forebrain and spinal cord tissue were extracted and 20 μg of protein was used for immunoblot analysis of SOD1 levels, using an antibody specific for human SOD1 (hSOD1) (a). Each lane represents an individual animal (n = 3). Graph represents densitometric quantification of hSOD1 levels normalized to GAPDH (b). Statistical analysis was conducted using GraphPad Prism (version 7.0 h) Error bars show mean ± S.D.; unpaired T-test. A.U.; arbitrary units. (TIF 248 kb) [file 13024_2018_253_MOESM12_ESM.tif]

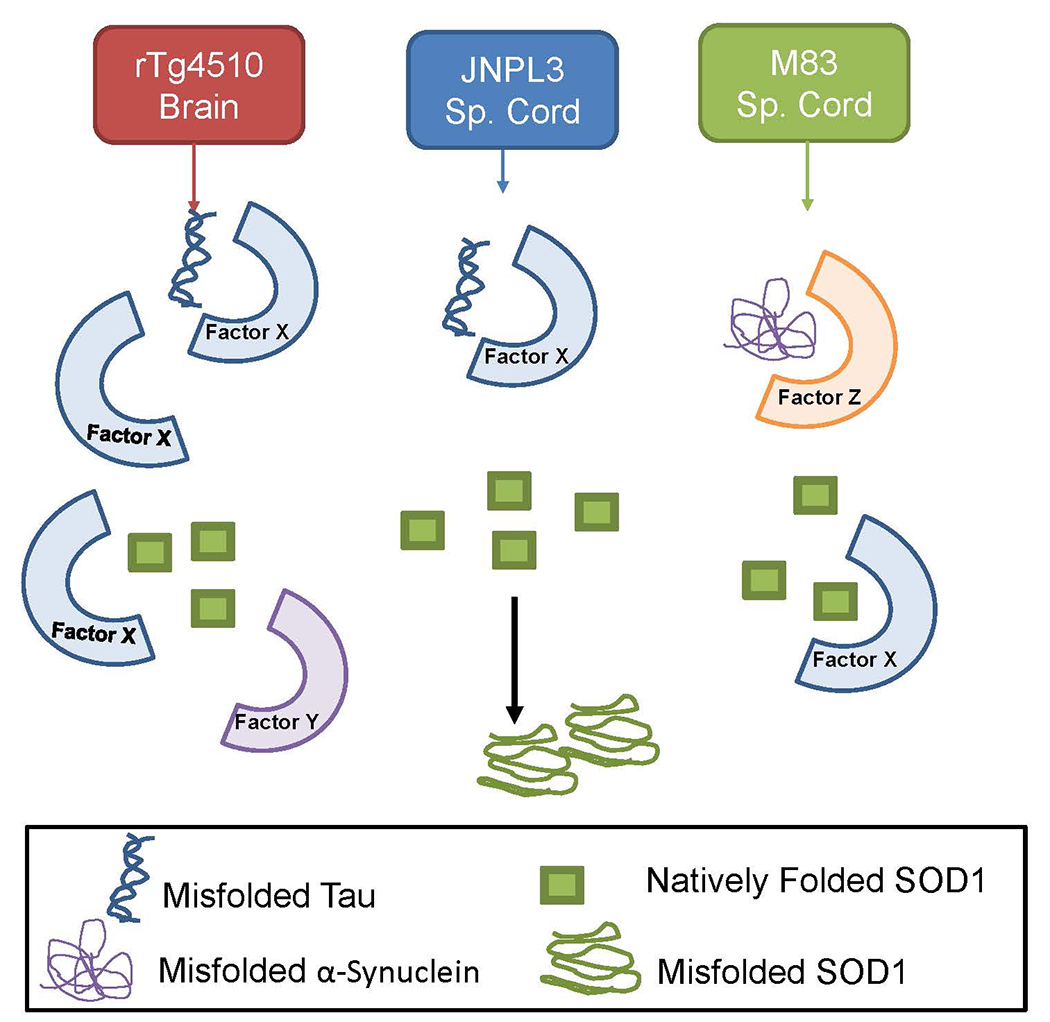

Supplement: Supplementary file 13 — Figure S12. Hypothetical mechanism of differential effects of tauopathy versus synucleinopathy on G85R-SOD1:YFP secondary aggregation in the spinal cord and cortex. In the JNPL3 spinal cord, misfolded tau occupies proteostatic factors (Factor X) that the mutant SOD1 reporter is also dependent upon for folding or degradation. In the cortex of rTg4510 mice, the levels of Factor X could be higher, or other proteostatic factors specific to brain (Factor Y) could be present to prevent the aggregation mutant SOD1. Meanwhile, misfolded αSyn occupies proteostatic factors distinct from those of tau (Factor Z), leaving a sufficient level of Factor X to prevent the aggregation of mutant SOD1. (TIF 553 kb) [file 13024_2018_253_MOESM13_ESM.tif]
